# Linking microbial community composition of three rootstock genotypes to apple replant disease susceptibility

**DOI:** 10.1186/s40793-026-00957-w

**Published:** 2026-09-08

**Authors:** Nils Orth, Nils Siefen, Jannika Staudt, Fatma M. Mahmoud, Sarah Benning, Silvia Gschwendtner, Tom P. Pielhop, Kristin Hauschild, Michael Schloter, Michaela Schmitz, Traud Winkelmann

**Affiliations:** 1 Institute of Plant Genetics, Leibniz University Hannover, Herrenhäuserstr. 2, 30419 Hannover, Germany; 2 Institute of Crop Sciences and Resource Conservation - Horticultural Science, University of Bonn, Auf dem Hügel 6, 53121 Bonn, Germany; 3 Competence Centre of Horticulture, DLR Rheinpfalz, Campus Klein-Altendorf 2, 53359 Rheinbach, Germany; 4 Department of Sustainable Food Systems, University of Applied Science Südwestfalen, Lübecker Ring 2, 59494 Soest, Germany; 5 Research Unit Comparative Microbiome Analysis, Helmholtz Munich, Ingolstädter Landstraße 1, 85764 Oberschleißheim, Germany; 6 Institute of Horticultural Production Systems, Leibniz University Hannover, Herrenhäuserstr. 2, 30419 Hannover, Germany; 7 Institute for Epidemiology and Pathogen Diagnostics, Julius Kühn Institute, Federal Research Centre for Cultivated Plants, Messeweg 11-12, 38104 Braunschweig, Germany

**Keywords:** Apple replant disease (ARD), Rhizosphere bacteria, Endophytic bacteria, Apple rootstock, *Streptomyces*, Metabarcoding, qPCR

## Abstract

**Background:**

Apple replant disease (ARD) is a worldwide issue of complex biotic origin. The exact aetiology has yet to be fully elucidated and environmentally friendly and economically feasible countermeasures are sparse. The breeding of tolerant apple rootstock genotypes shows some promise as reduced sensitivity on selected ARD-affected soils has been shown for some rootstock genotypes. By adding insights into the root-associated microbiome, we aimed to complement previously published results, examining the response in growth, gene expression and production of phenolic compounds in roots of seven *Malus* genotypes in ARD soil. We selected the two least susceptible rootstock genotypes of the previous study, EMR.2 and G.935, in addition to the susceptible rootstock genotype M.26. The objective of this study was to gain insights into the bacterial community composition of the rhizosphere and root endosphere of less susceptible rootstock genotypes in comparison to the susceptible one.

**Results:**

In an Illumina-based sequencing approach, the composition of rhizosphere as well as endophytic bacterial communities was investigated for apple plants in ARD soil and disinfected ARD soil. ARD impacted the rhizosphere stronger than the endosphere, where rootstock genotype influence was stronger. G.935 generally showed a more distinct and diverse microbiome than the other two rootstock genotypes. In bacterial communities, the relative abundance of *Pseudomonadota* decreased in ARD samples, while *Actinomycetota* increased. In the endosphere, this was mostly accounted for by *Streptomyces*. A qPCR approach confirmed the high abundance of *Streptomyces*. Interestingly, another qPCR approach did not indicate a high abundance of plant pathogenic *Streptomyces* in the same samples.

**Conclusions:**

ARD strongly influenced the bacterial microbiome in the rhizosphere and, to a lesser extent, the root endophytic microbiome. The distinct and more diverse bacterial microbiome of G.935 might help against biotic stress, such as ARD. However, EMR.2 was previously found to be less susceptible to ARD, too, suggesting additional factors must play a role and additional research is necessary. Pathogenicity of *Streptomyces* could not be proven but repeatedly high abundance of root endophytic *Streptomyces* under ARD conditions indicate an important role.

**Supplementary Information:**

The online version contains supplementary material available at https://doi.org/10.1186/s40793-026-00957-w.

## Highlights

The endophytic microbial communities of two East Malling rootstocks M.26 and EMR.2 are more similar to each other than to that of the Geneva rootstock G.935.

Endophytic *Streptomyces* appear at high relative and absolute abundances in roots from ARD soil, regardless of rootstock genotype.

qPCR results suggest that these endophytic *Streptomyces* are likely not plant pathogenic.

**Supplementary Information:**

The online version contains supplementary material available at https://doi.org/10.1186/s40793-026-00957-w.

## Introduction

Apple replant disease (ARD) describes a dysfunctional biotic state of a soil for the growth of apple plants caused by previous cultivation of apple [1]. Roots of affected trees are damaged and symptoms usually include blackening, reduced number of fine roots and root tip necrosis, among others [2]. Aboveground, the disease leads to stunted growth as well as reduced yield and fruit quality [1, 3], negatively impacting tree nurseries and orchards alike.

The exact aetiology of ARD is not fully understood yet but biotic factors are accepted as the driving force of the disease. A number of pathogens have been identified to contribute to the disease. Among them are fungi, such as members of *Ilyonectria*, *Dactylonectria*, *Cylindrocarpon*, *Fusarium*, *Rhizoctonia* and *Nectria*, and oomycetes, such as members of *Pythium* and *Phytophthora* [4–10]. Furthermore, nematodes, such as plant pathogenic *Pratylenchus penetrans* as well as free-living nematodes, have been associated with ARD [11–13]. To date, no bacteria found in conjunction with ARD have been proven to be pathogenic. However, dramatic differences in the bacterial community composition between samples from healthy and ARD-affected soil have been observed in bulk soil, rhizosphere and root endosphere [4, 14–18]. Therefore, it has been proposed that, alongside pathogenic pressure, alterations to the microbiome may also contribute to the disease [14].

*Actinomycetota* (formerly denominated as *Actinobacteria*) specifically have been found in high abundance in ARD-affected apple roots compared to those from healthy soils [2, 19–21]. Sequencing-based approaches revealed members of the genus *Streptomyces* to be largely responsible for this difference [22]. However, despite their high relative abundance, isolating strains has been proven difficult in the past [15, 20]. So far, only Tewoldemedhin et al. (2011) [23] successfully obtained several *Streptomyces* isolates from ARD-affected roots. However, they turned out to be non-pathogenic in subsequent tests.

Currently, effective management strategies to cope with ARD mostly rely on soil fumigation with chemical disinfectants [24–26]. However, due to their negative environmental impact, they are being phased out in some regions of the world, i.e. the EU [27]. Yet, alternative management strategies are often economically unfeasible or lack efficacy [1]. Breeding of tolerant rootstock genotypes could potentially provide an environmentally-friendly solution [28–30] that comes without considerable additional workload or expenses for the farmers. This strategy shows some promise, as reduced susceptibility toward ARD has been shown to exist in the *Malus* germplasm [31]. Furthermore, potential ARD-tolerance was postulated for several Geneva rootstock genotypes [28, 32, 33]. However, the relationship between rootstock genotype, microbial community composition, and ARD tolerance has not been systematically investigated.

**Fig. 1:**
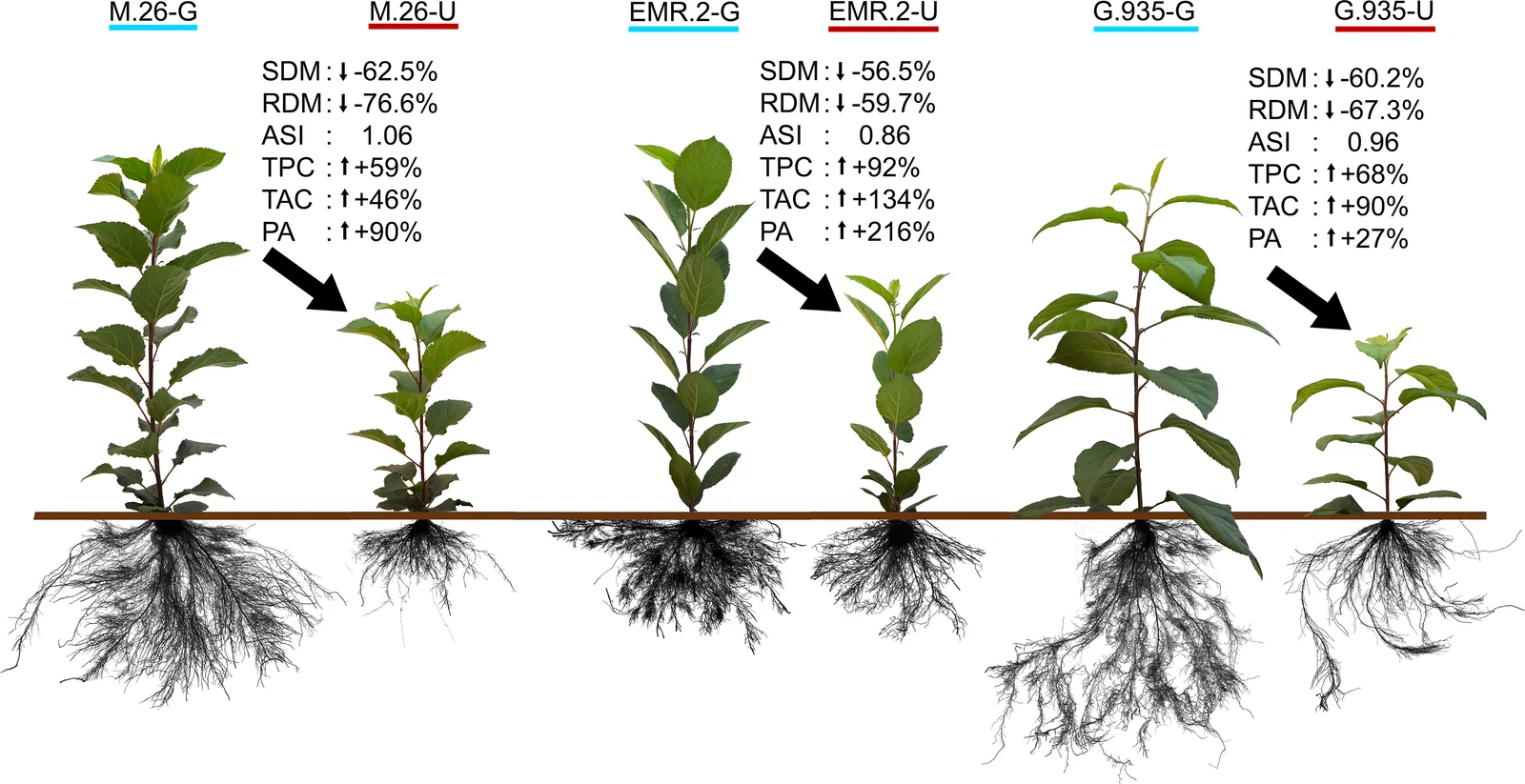
Representative apple plants of the three rootstock genotypes, M.26, EMR.2, and G.935, after 8 weeks of growth in either ARD soil (U) or gamma-irradiated soil (G). The main findings published by Siefen et al. (2024) [34] showing the differences between U and G are displayed for each genotype: shoot dry mass (SDM), root dry mass (RDM), ARD susceptibility index (ASI; higher values indicate higher susceptibility), total phenolic content (TPC), total antioxidant capacity (TAC), and phytoalexin content (PA). (Modification of previously published Fig. 1 [34])

Our study is based on the experiment conducted and published by Siefen et al. (2024) [34], the results of which are partly summarised in Fig. 1. In brief, they compared growth parameters and the physiological response in terms of production of phytoalexins and other phenolic compounds of seven *Malus* genotypes in order to identify their ARD susceptibility. Therefore, in vitro propagated young plants were cultivated in a severely ARD-affected soil as well as in ARD-affected soil disinfected by gamma-irradiation. The ARD susceptibility was classified based on the growth difference in both soil variants.

Here, we used a molecular barcoding approach to gain insights into the bacterial community structure in rhizosphere and root endosphere of different apple rootstock genotypes. We aimed to unravel whether moderately less susceptible rootstock genotypes shape their bacterial communities in these compartments more strongly and thus lessen the impact of ARD. Our main research question was how ARD and rootstock genotype affect bacterial community composition and diversity in the root endosphere and rhizosphere. An additional research question concerned the role of *Streptomyces* in ARD, including their potential plant pathogenicity. Here, we used a qPCR-based quantification to confirm if high relative abundances of *Streptomyces*, detected by molecular barcoding, can be attributed to an increase in absolute abundance of *Streptomyces* rather than a decrease of the overall bacteria population. A second qPCR approach was employed to address potential plant pathogenicity of the detected *Streptomyces*. For the second question, we hypothesised that (i) the accumulation of endophytic *Streptomyces* in ARD-affected roots is lower in potentially tolerant rootstocks; and that (ii) plant pathogenic *Streptomyces* strains with the *txtAB* gene are enriched in ARD-affected roots.

## Material & methods

### Plant and soil material & experimental setup

The samples used in this study were collected from the experiment conducted by Siefen et al. (2024) [34]. A detailed description of the experimental setup, including the acquisition of plant material, soil handling and greenhouse biotest, can be found in the aforementioned publication. Briefly, six *Malus* rootstock genotypes (M.26, EMR.2, M.200, G.935, G.214, Selection 4) and one wild type accession from *M. sargentii* (MAL0739) were propagated and rooted in vitro, and acclimatised in peat substrate to greenhouse conditions before the start of the experiment. Of these seven *Malus* genotypes, three were selected for examination of their microbiome based upon their ARD susceptibility indices (ASIs, Fig. 1): the susceptible rootstock genotype M.26 and the two genotypes EMR.2 and G.935, which were classified as less susceptible in this experiment [34].

ARD-affected soil for the greenhouse experiment had been collected from the ORDIAmur reference site in Heidgraben (53°41’57.5"N, 9°40’59.6"E, Schleswig-Holstein, Germany) in January 2020. A detailed description of the soil parameters has previously been published by Mahnkopp et al. (2018) [35]. The soil was sieved with a 5 mm mesh and half of the soil was disinfected by gamma-irradiation (Synergy Health Radeberg GmbH, Radeberg, Germany), which served as a control (G), while the other half remained untreated (U). Successful disinfection was confirmed via CFU counting from dilution plating of both soil treatments.

The soils were fertilised with 2 g/L Osmocote Exact 3-4 M (ICL Specialty Fertilizers, Geldermalsen, The Netherlands) prior to planting. Each plant was planted into a single pot with 1.5 L soil. In total, 18 plants of each genotype were planted per soil treatment (G and U). All plants were cultivated in a greenhouse for eight weeks (drip irrigation, mean temperature 22.4 ± 3.8 °C, mean relative air humidity 44.9 ± 17.5%, 16/8 photo period, average photosynthetically active radiation 103 µmol m^2^ s^− 1^).

Per genotype (M.26, EMR.2 & G.935), six representative plants of each sampling time point and soil treatment were selected for sampling: i.e. before planting (T0), uprooted plantlets, freed from the peat substrate used for acclimatisation, and after eight weeks six pots with plants grown in untreated (U) and gamma-irradiated (G) soil. For ease of reading, sampling points and soil treatments will be collectively referred to as treatments in the subsequent text. Rhizosphere samples were only collected after eight weeks: Of each plant, the bulk soil was removed carefully by hand and 1–2 mL of the remaining, root-adhering soil was collected in 2 mL reaction tubes and stored at -80 °C until DNA extraction. Afterwards, the root system was carefully washed with tap water and a representative mix of roots was cut off and collected in a 15 mL centrifuge tube. A few droplets of tap water were added to ensure 100% humidity. To minimise impact of physiological processes, the tubes were stored in the dark at 4 °C for a maximum of 72 h until surface disinfection. Surface disinfection was done according to Mahnkopp-Dirks et al. (2021) [22] and surface disinfected roots were stored at -80 °C until DNA extraction.

### DNA extraction

The DNA from 0.5 g rhizosphere per sample was extracted following the protocol described by Stempfhuber et al. (2017) [36], which is a modified protocol of the originally developed one by Lueders et al. (2004) [37]. For DNA extraction from roots, 60 mg of frozen root material was homogenised in 2 mL tubes with two sterile metal beads each, using a Retsch mill (RETSCH GmbH, Haan, Germany) at 27 s^− 1^ for 30 s. DNA extraction from the homogenised root samples was done with the Invisorb Spin Plant Mini Kit (Invitek Molecular, Berlin, Germany) according to the manufacturer’s instructions with two modifications: An additional centrifugation step (11,000 *g*, 7 min) was added prior to pre-filtration to avoid clogging of the filter. Also, the elution procedure was performed in two steps with 50 µL elution buffer each rather than in one step with 100 µL elution buffer to increase yield.

### Short read sequencing

Illumina Miseq sequencing of rhizosphere and endophytic bacteria was performed at the Research Unit Comparative Microbiome Analysis (Helmholtz Munich, Germany). The bacterial rhizosphere community was assessed by using the primers 515F [38] and 806R [39] to amplify the V4 region of the *16S rRNA* gene, according to the protocol described by Orth et al. (2024) [40] with the following modifications: The PCR reaction mixtures contained 0.3 µL (10 pmol) of each primer (instead of 0.5 µL) and 17 ng DNA (instead of 15 ng). For bacterial root endophytes, the primer pair 335F/769R [41] was used to amplify bacterial fragment of the *16S rRNA* gene (V3-V4 region) while excluding fragments derived from chloroplasts and mitochondria. The protocol used was previously described by Mahnkopp-Dirks et al. 2022 [15] and modified as follows: The PCR reaction (5 min initial denaturation at 98 °C; 30 cycles of 10 s denaturation at 98 °C, 30 s annealing at 60 °C and 30 s elongation at 72 °C; 5 min final elongation at 72 °C) was done with 15 ng DNA, 12.5 µL NEBnext high fidelity polymerase (New England Biolabs, Ipswich, USA) and 5 pmol of each primer (25 µL total reaction volume). PCR blank (H_2_O) controls were also sequenced and resulting reads were used to prefilter ASV tables obtained from sample sequencing. The fungal root endophyte community was also assessed. However, due to low numbers of quality reads, the data was moved to the supplements. A short section, including Material & Methods, Results and Discussion regarding the fungal endophytes can be found in supplementary material (section “Analyses of Fungal Endophytes”).

Amplicon sequence variants (ASVs) were analysed using the QIIME 2 software package release 2019.10 with default parameters [42]. FASTQ files were trimmed and merged with a minimum read length of 50 and minimum Phred score of 15 using AdapterRemoval [43]. Quality control was done using the QIIME 2 plugin DADA2 by removing 10 bp n-terminally [44]. For bacteria, reads were truncated at position 280 (forward) and 210 (reverse), respectively, with an expected error rate of 6. For taxonomic analysis, Naive Bayes classifier was pre-trained on the SILVA_132 QIIME release 99% for bacterial assignment. All ASVs not assigned at genus level or higher were annotated as “unassigned X” (X being the next highest assigned taxon).

The following procedure was done on both data sets (rhizosphere bacteria and endophytic bacteria). Rarefaction curves were created in RStudio ver. 2025.05.0 [45] based on R version 4.5.1 [46] with the “rarefy()” function of the vegan-package [47]. For rhizosphere bacteria, one sample was removed to ensure decent saturation of all curves at the cut-off (Suppl. Figure 1). Raw count data was rarefied by random subsampling of the lowest number of reads in a sample: 14,401 reads for rhizosphere bacteria, 13,370 reads for bacterial endophytes. α-diversity indices (Observed species, Simpson (1-*D*)) were determined with the “estimate_richness()” function of the phyloseq package [48]. A Principal Coordinate Analysis (PCoA, Bray-Curtis) was performed to calculate the β-diversity using the “vegdist()” function and visualised via the ordiplot() function of the vegan package [47]. Taxa were summed using the “Pivot” function in Excel 2016 (Microsoft, Redmond, USA). Relative abundances (RA) were calculated and the core microbiome at phylum level was filtered as follows: at least 1% RA in all samples of one genotype-treatment combination. Bar plots at phylum level were done in Excel (Microsoft, Redmond, USA). Venn-Diagrams were created using the web tool provided by the Bioinformatics Institute Ghent [49] and further modified with Inkscape version 0.92.4 [50].

### Quantification of *Streptomyces* via quantitative PCR

A qPCR protocol for the quantification of *Streptomyces* in apple roots was adapted from the method originally developed by Rintala & Nevalainen (2006) [51]. The primer pair SMfw8/SMrev9 amplifies a fragment of the *23S rRNA* gene and reliably only targets DNA from *Streptomyces* spp (Suppl. Table 1). Plasmid-DNA from an *Escherichia coli* transformed with a 71 bp sequence of *Streptomyces pulveraceus* ES16 served as a positive control. DNA of an endophytic *Novosphingobium sp.* served as the negative control. All qPCRs were conducted using a CFX Connect™ cycler (Bio-Rad, Hercules, USA) with the following settings: initial polymerase activation (2 min at 50 °C), initial denaturation (10 min at 95 °C), 40 cycles of denaturation (15 s at 95 °C) followed by alignment and elongation (1 min at 58 °C). A melting curve from 65 °C to 95 °C (0.5 °C increments) was recorded at the end. A detailed protocol can be found in the supplementary material (section “Comprehensive qPCR Material and Methods”). The data was recorded with CFX Maestro software version 2.3 (Bio-Rad, Hercules, USA). Log-transformation and a bar plot were facilitated in Excel (Microsoft, Redmond, USA). Per sample, the log-transformed data was also plotted against the RA of *Streptomyces* (sum of all ASVs, see Section “Short read sequencing”) and a linear regression was calculated.

A qPCR protocol established by Qu et al. (2008) [52] to quantify pathogenic *Streptomyces* spp. was adapted for root material from apples. DNA extraction for the positive control (*Streptomyces scabies* 87.22, NC_013929.1) and negative control (*Streptomyces venezuelae* NRRL B-65442, NZ_CP018074.1), both kindly provided by the Institute of Microbiology (Leibniz University Hannover, Germany), was done as described in the supplementary material for *Novosphingobium* sp. The qPCRs were performed in a CFX Connect cycler (Bio-Rad, Hercules, USA) with the following settings: initial denaturation (15 min at 95 °C), 45 cycles of denaturation (15 s at 95 °C), annealing (30 s at 60 °C) and elongation (30 s at 72 °C). A melting curve was recorded between 65 °C and 95 °C (0.5 °C increments). A detailed protocol can be found in the supplementary material (section “Comprehensive qPCR Material and Methods”).

### Statistics

Statistical analyses were performed in RStudio version 2025.05.0 [45] based on R version 4.5.1 [46]. For α-diversity indices, data distribution was first assessed for normality. When normally distributed, an ANOVA was conducted using the base R function “aov()”, followed by a Tukey’s HSD post-hoc test using the “HSD.test()” function of the agricolae package (de Mendiburu, 2021) [53]. When normality was not met, a Wilcoxon test was performed using the “wilcox_test()” function of the rstatix package [54]. A PERMANOVA was performed for the β-diversity data using the “adonis2()” of the vegan package [47]. Differences of the relative abundance at phylum and ASV level were determined pairwise with the “DESeq()” function of the DESeq2-package [55]. To validate the results from DESeq2, ALDEx2 was also performed in R. Prior to this analysis, taxa were filtered to reduce sparsity by retaining only ASV with counts ≥ 10 (endophytes) and ≥ 50 (rhizosphere) in at least 20% of samples. Centered log-ratio (CLR) transformation was performed using aldex.clr with 128 Monte Carlo instances and the “all” denominator setting. Pairwise differential abundance analyses were performed between treatment groups within each genotype using Wilcoxon rank sum test combined with effect size estimation (aldex.effect). The log-transformed data from the qPCR was fitted via the “lm()” base R function and a two-way comparison of genotype and treatment was calculated via the “emmeans()” function of the emmeans package [56].

## Results

### Microbial diversity was influenced by genotype and treatment

**Fig. 2:**
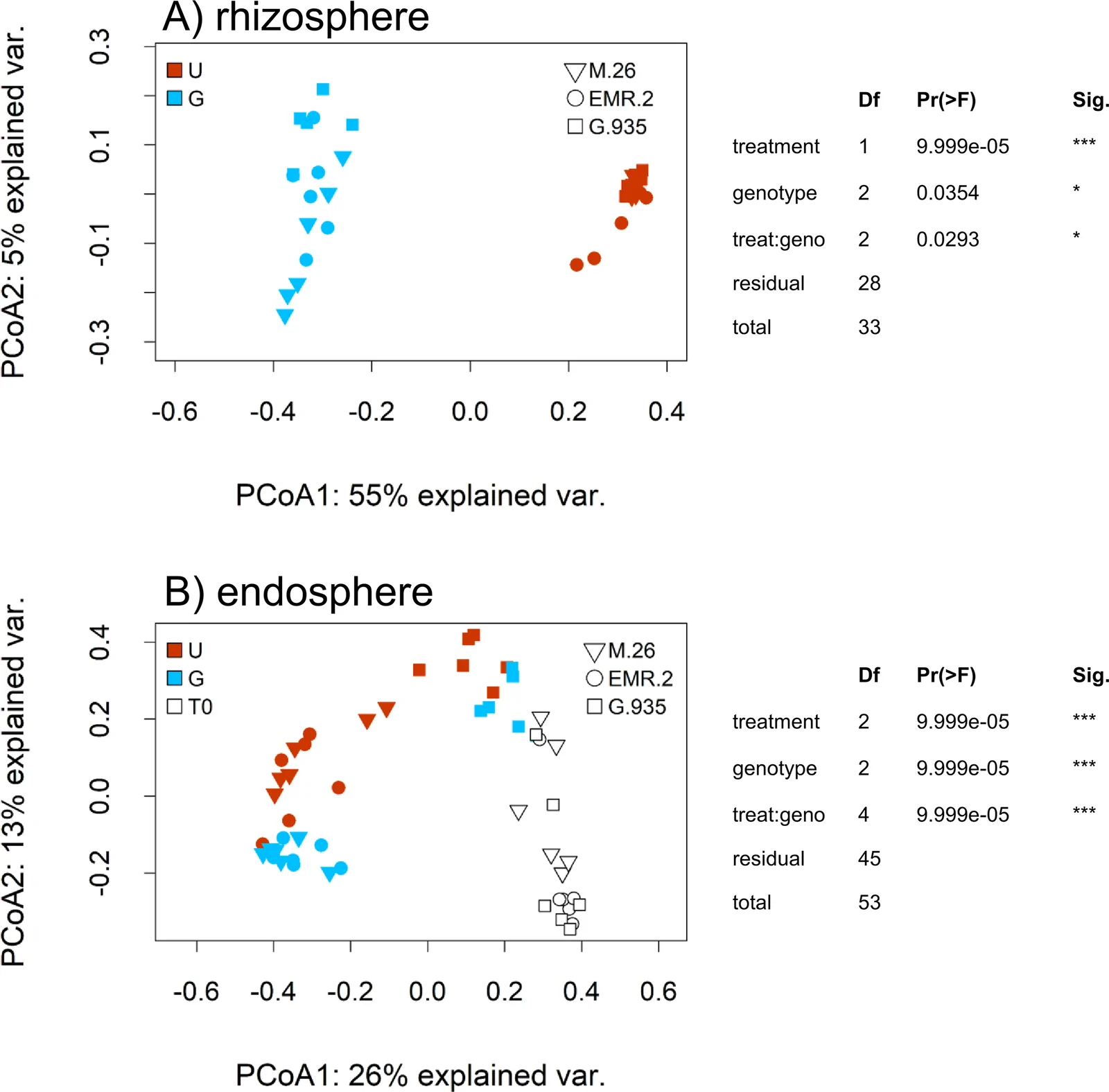
β-diversity displayed by Principal Coordinate Analysis (PCoA) of (**A**) rhizosphere bacteria and (**B**) root endophytic bacteria for apple rootstock genotypes M.26, EMR.2, and G.935 before planting (T0) and after 8 weeks of growth in either apple replant disease-affected (U) or gamma-irradiated (G) soil. Significant effects of treatment, genotype and their interaction were determined by a PERMANOVA (*n* = 5–6, * *p* < 0.05, *** *p* < 0.001)

In the rhizosphere, the number of observed bacterial genera was significantly higher in ARD soil (U) compared to gamma-irradiated soil (G) for the rootstock genotypes EMR.2 and G.935, but not for M.26 (Table 1a). The Simpson indices were at the upper limit of 1 across all rootstock genotypes, regardless of soil treatment, and did not differ significantly from each other. No significant differences between the three rootstock genotypes within one treatment, either ARD soil (U) or gamma-irradiated soil (G) were determined. A Principal Coordinate Analysis (PCoA) displayed clustering mostly based on soil treatment, i.e. samples from ARD (U) or gamma-irradiated (G) soil clustered together, respectively (Fig. 2a). However, significant differences between genotypes and a treatment-genotype interaction were also found as indicated by the PERMANOVA output (Fig. 2a).

As expected, the number of observed endophytic bacterial genera was generally lower than that in the rhizosphere and no significant differences were determined, neither among treatments nor among genotypes (Table 1b). PERMANOVA and clustering (Fig. 2b) showed clear differences between genotypes and soil treatments. For all three genotypes, distinct clustering was observed for T0, G, and U. Within both soil treatments (G and U), G.935 formed a separate cluster, while M.26 and EMR.2 clustered closely together.

**Table 1:**
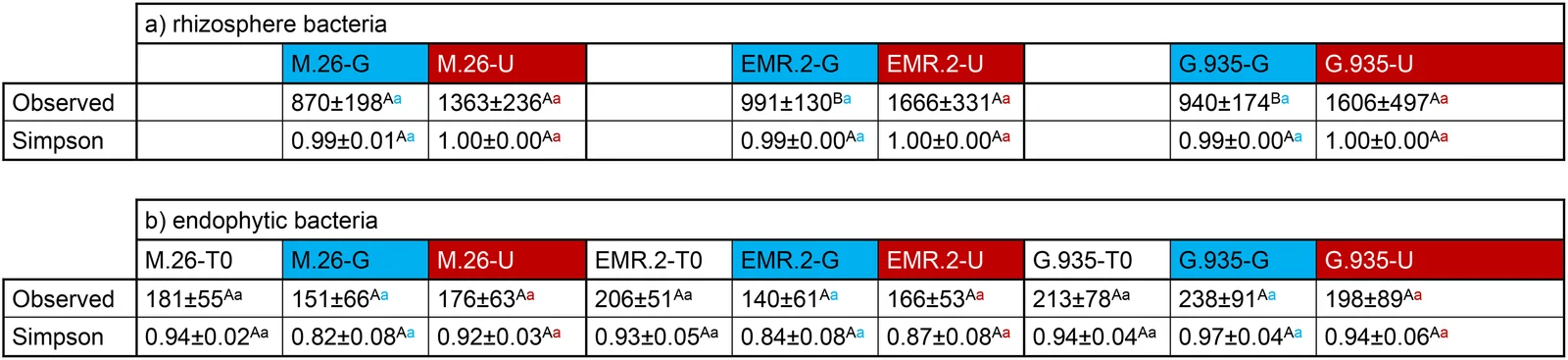
Mean α-diversity indices “Observed” and “Simpson” (1-*D*) with standard deviation of a) rhizosphere bacteria and b) root endophytic bacteria of apple rootstock genotypes M.26, EMR.2, and G.935 before planting (T0) and after 8 weeks of growth in either apple replant disease-affected (U) or gamma-irradiated (G) ARD-affected soil. Differing lower case letters indicate significant differences between genotypes within the same treatment, i.e. T0 (black), G (blue) or U (red). Statistical differences were determined with either Tukey’s test (Observed) or Wilcox’ test (Simpson). Differing upper case letters indicate significant differences between the treatments within one genotype, i.e. M.26, EMR.2 or G.935 (*n* = 5–6, *p* < 0.05)

### Microbial communities were shaped more by treatment than plant genotype at phylum level

**Fig. 3:**
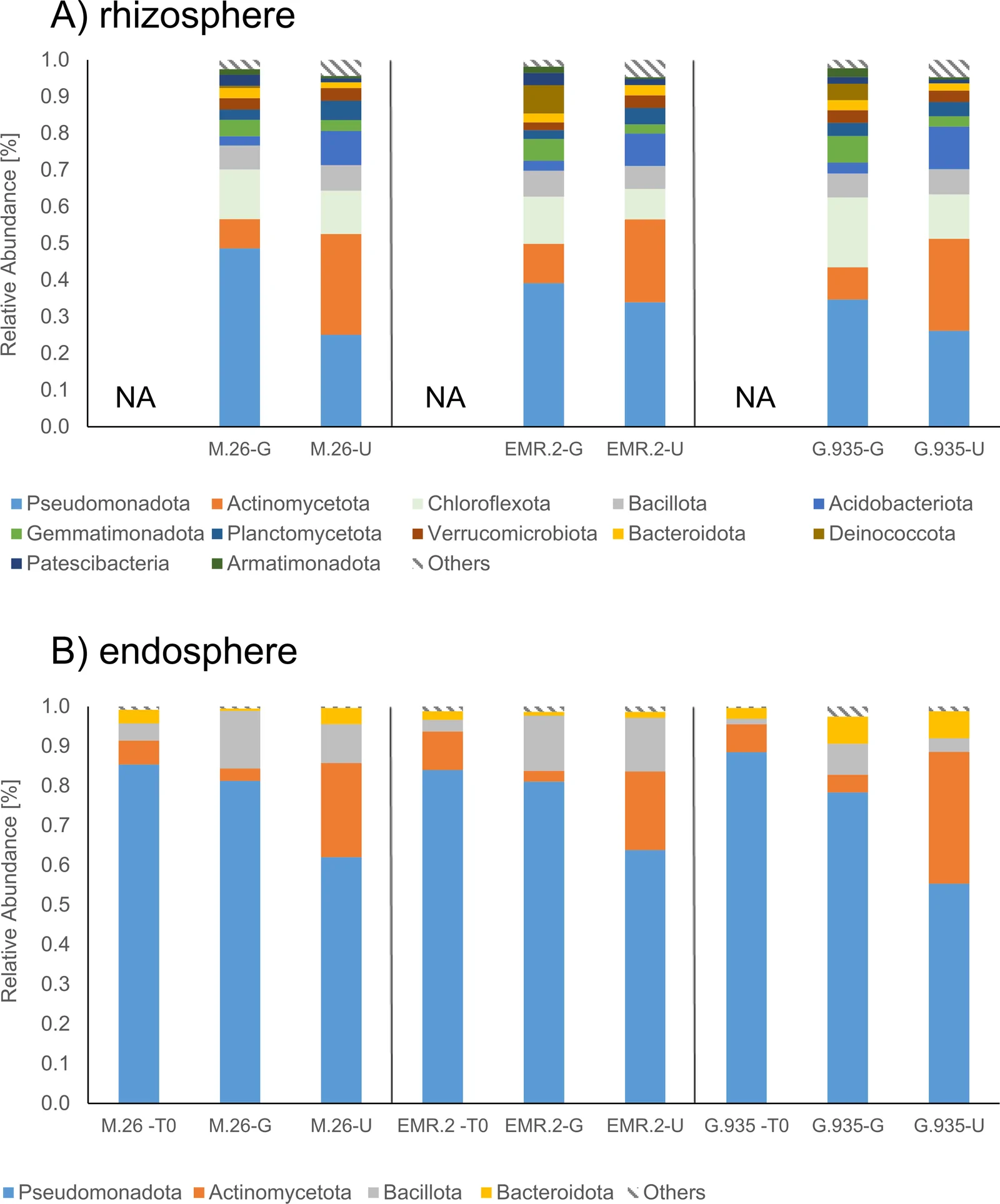
Relative abundance (RA, %) at phylum level of (**a**) rhizosphere bacteria (RA > 1%, 100% frequency, NA = samples not available) and (**b**) endophytic bacteria (RA > 1%, 100% frequency) for the apple rootstock genotypes M.26, EMR.2, and G.935 before planting (T0) and after 8 weeks of growth in either apple replant disease-affected (U) or gamma-irradiated (G) soil. Statistically significant differences are displayed in Suppl. Table 2 (rhizosphere bacteria) and Suppl. Table 3 (endophytic bacteria)

Differential abundances between different taxa were tested with DESeq2 and validated with ALDEx2. In the rhizosphere, a significant reduction of *Pseudomonadota* (previously *Proteobacteria*) in ARD soil (U) compared to the gamma-irradiated soil (G) was observed across all three rootstock genotypes (Fig. 3a, Suppl. Table 2). For M.26, the relative abundance (RA) decreased from 48.6 ± 1.1% in G to 25.1 ± 1.1% in U, while a less pronounced decrease from 39.1 ± 0.5% (G) to 33.9 ± 2.0 (U) and from 34.7% (G) to 26.2 ± 0.8% was observed for EMR.2 and G.935, respectively. The opposite was observed for *Actinomycetota*: RAs in rhizosphere of M.26 significantly increased from 8.0 ± 1.1% to 27.5 ± 6.4% in U compared to G, from 10.7 ± 1.9% (G) to 22.6 ± 5.4% (U) in rhizosphere of EMR.2, and from 8.8 ± 1.7% (G) to 25.1 ± 1.8% (U) in rhizosphere of G.935. Genotypic differences barely existed on the level of bacterial phyla, with no significant differences between M.26 and EMR.2, and only a few differences between G.935 and either M.26 or EMR.2 (Supp. Table 2).

Also in the root endosphere, *Pseudomonadota* had the highest RA on phylum level across all rootstock genotypes and treatments (Fig. 2b, Suppl. Table 3). Corresponding to the RA data of the rhizosphere, the RA of *Pseudomonadota* was significantly lower in roots grown in U compared to G, while the RA of *Actinomycetota* was substantially higher in roots grown in U compared to G. However, due to high variation between individual samples, only the difference in the RA of *Actinomycetota* between G.935-G (4.4 ± 1.8%) and G.935-U (33.2 ± 29.7%) was significant. Genotypic differences on the level of bacterial phyla were again only observed between G.935 and the other two rootstock genotypes.

### Dynamics of single ASVs in response to treatment, plant genotype and time point

#### Rhizosphere bacteria

**Fig. 4:**
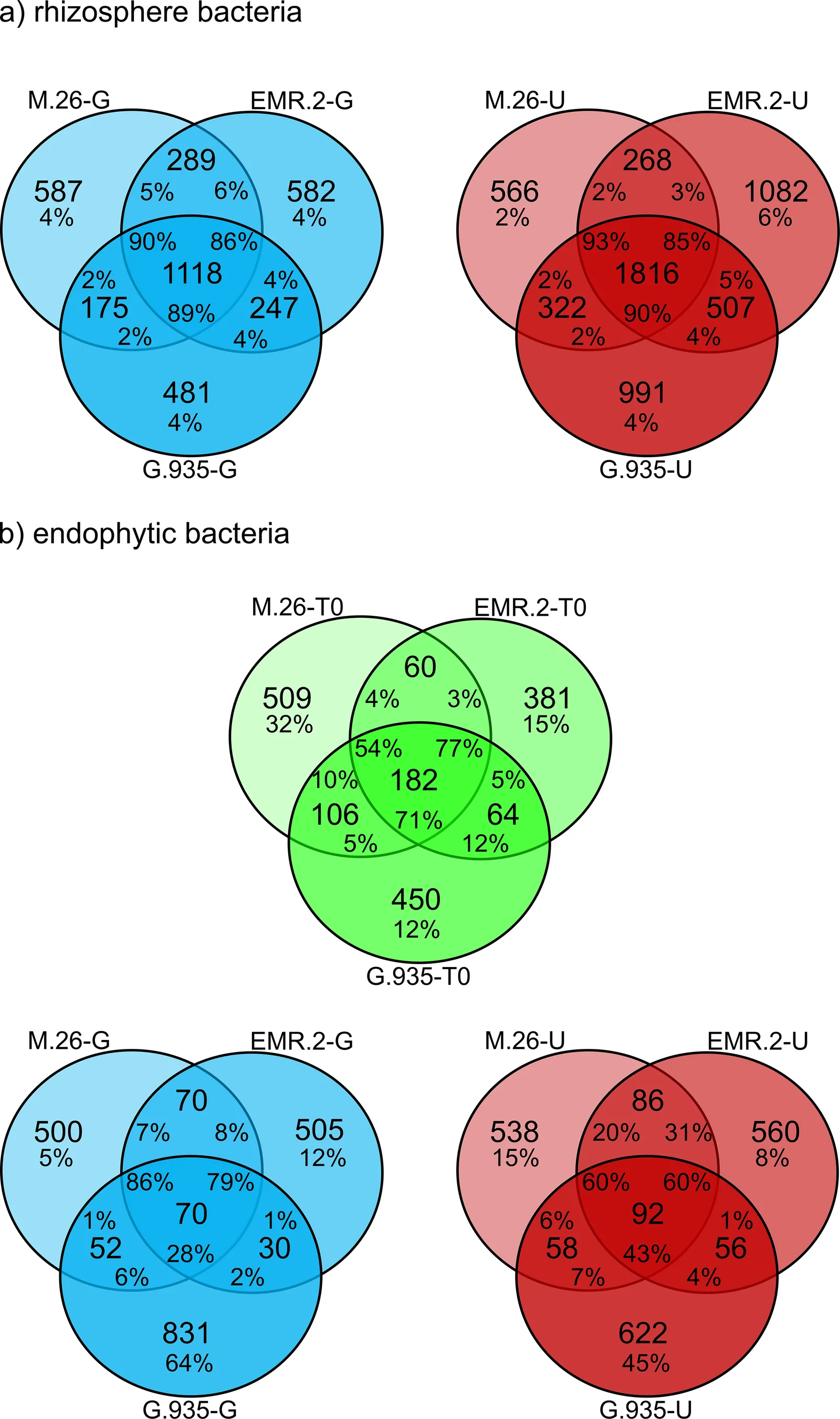
Venn-Diagrams comparing the ASV numbers and their summed relative abundances (%) of (**a**) rhizosphere bacteria and (**b**) endophytic bacteria between the apple rootstock genotypes M.26, EMR.2, and G.935 before planting (T0) and after 8 weeks of growth in either apple replant disease-affected (U) or gamma-irradiated (G) ARD soil. The percentages are aligned towards the rootstock genotype they belong to (e.g. M.26 aligned towards top left) and show the contribution of ASVs of the respective compartment to the total RA of each rootstock genotype

In the rhizosphere of plants grown in gamma-irradiated (G) soils, 587, 582, and 481 unique ASVs were detected for M.26, EMR.2, and G.935, respectively (Fig. 4a). However, these unique ASVs accounted for only about 4% of the total relative abundance (RA) in each genotype. In contrast, 1,118 ASVs were shared among all three rootstocks and comprised the majority of the bacterial community, contributing 86% − 90% of the total RA. A similar pattern was observed in the untreated (U) soil, where ASVs unique to a single rootstock contributed only 2% − 6% of total RA.

**Fig. 5:**
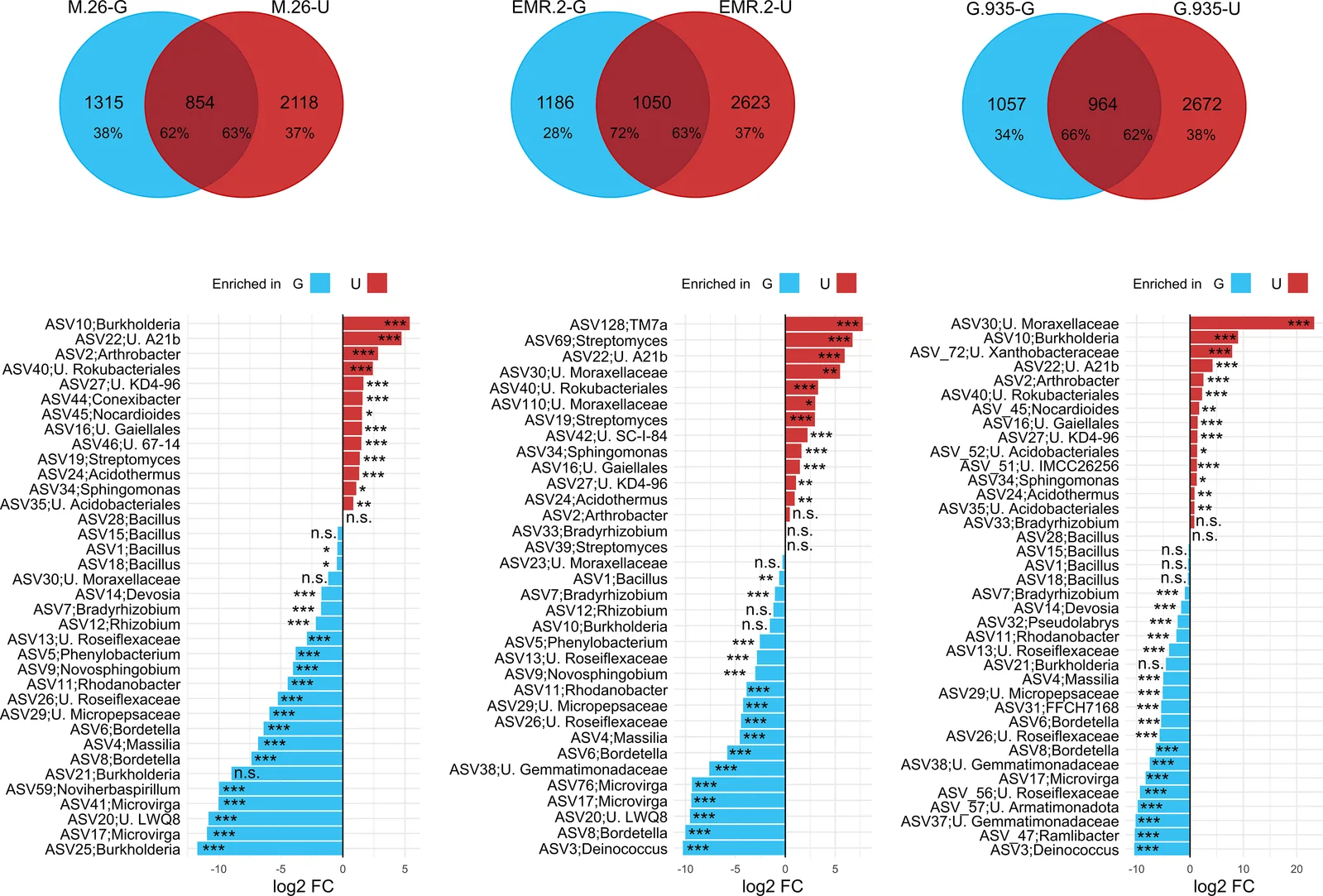
Venn-Diagrams comparing the numbers and relative abundance (RA, %) of rhizosphere bacteria at ASV level from gamma-irradiated ARD soil (G) and untreated ARD soil (U) of each rootstock genotype (M.26, EMR.2 and G.935) separately. The percentages are aligned towards the treatment they belong to, i.e. G towards left and U towards right, and show the contribution of each compartment to the total RA. Below, log2 fold changes (log2 FC) of the top 20 ASVs of G and U for each of the three rootstock genotypes are displayed (DESeq2; *n* = 5–6; ‘*p ≤ 0.05’, ‘**p ≤ 0.01’, ‘***p ≤ 0.001)

To explore treatment effects, rhizosphere bacterial communities were compared between the G and U variants for each rootstock genotype (Fig. 5, Suppl. Table 4). For M.26, 1,315 ASVs were unique to G, 2,118 were unique to U, and 854 ASVs were shared between both treatments, which represented two-thirds of total RA. Comparable distributions were observed for EMR.2 and G.935.

Analysis of the 20 most abundant ASVs further illustrated these patterns.

For M.26, five ASVs were unique to the G treatment (*Microvirga* (ASV17), *Burkholderia* (ASV25, ASV21), *Noviherbaspirillum* (ASV59), and an unclassified *LWQ8* (ASV20)). Among ASVs with > 1% RA, *Massilia*, *Phenylobacterium*, *Bordetella*, *Bradyrhizobium*, *Novosphingobium*, *Rhodanobacter*, *Rhizobium*, and unclassified *Roseiflexaceae* were all significantly more abundant in M.26-G than in M.26-U, whereas only *Bacillus* (ASV1) and *Arthrobacter* (ASV2) were significantly higher in RA in M.26-U.

For EMR.2, only two *Microvirga* ASVs (ASV17, ASV76) were unique to G. Among the dominant taxa (> 1% RA), *Bacillus*, *Deinococcus*, *Massilia*, *Phenylobacterium*, *Bordetella*, *Bradyrhizobium*, *Novosphingobium*, *Burkholderia*, *Rhodanobacter*, *Rhizobium*, and unclassified *LWQ8* (ASV20) were significantly more abundant in EMR.2-G than in EMR.2-U. In contrast, two *Streptomyces* ASVs (ASV19, ASV69) were significantly more abundant in EMR.2-U.

In G.935, three ASVs (*Gemmatimonadaceae*, *Ramlibacter*, and unclassified *Amatimonadota*) appeared exclusively in G samples, while *Burkholderia*, *Moraxellaceae*, and *Xanthobacteraceae* were unique to U. Only *Arthrobacter* (ASV2) and unclassified *A21b* (ASV22) exceeded 1% RA and were significantly higher in G.935-U. Conversely, *Deinococcus*, *Massilia*, *Phenylobacterium*, *Bordetella*, unclassified *Roseiflexaceae*, *Microvirga*, and *Burkholderia* ASVs were significantly higher in G.935-G, each exceeding 1% RA.

Overall, the presence or absence of ARD significantly impacted the occurrence and RA of ASVs while differences between the examined rootstock genotypes were miniscule.

#### Root endophytic bacteria

At T0, the root endophytic bacterial community consisted of 509 unique ASVs in M.26 (32% of total RA), 381 ASVs in EMR.2 (15% RA), and 450 ASVs in G.935 (12% RA) (Fig. 4b). Only 182 ASVs were shared among all three rootstocks, yet these accounted for the majority of the total RA (54% − 77%).

After eight weeks of growth in gamma-irradiated (G) or untreated (U) ARD-affected soil, the ASV distribution shifted markedly (Fig. 4b). In the G treatment, M.26 and EMR.2 roots harboured 500 and 505 unique ASVs, corresponding to 5% and 12% of total RA, respectively, whereas G.935 contained 831 unique ASVs representing 64% of the total RA. Only 70 ASVs were shared among all three genotypes, but their contribution to total RA varied strongly: 86% in M.26, 79% in EMR.2, and 28% in G.935. A similar, though less pronounced, pattern was seen in the U treatment. Notably, 86 ASVs were shared exclusively between M.26 and EMR.2 while the overlap with G.935 remained minimal.

**Fig. 6:**
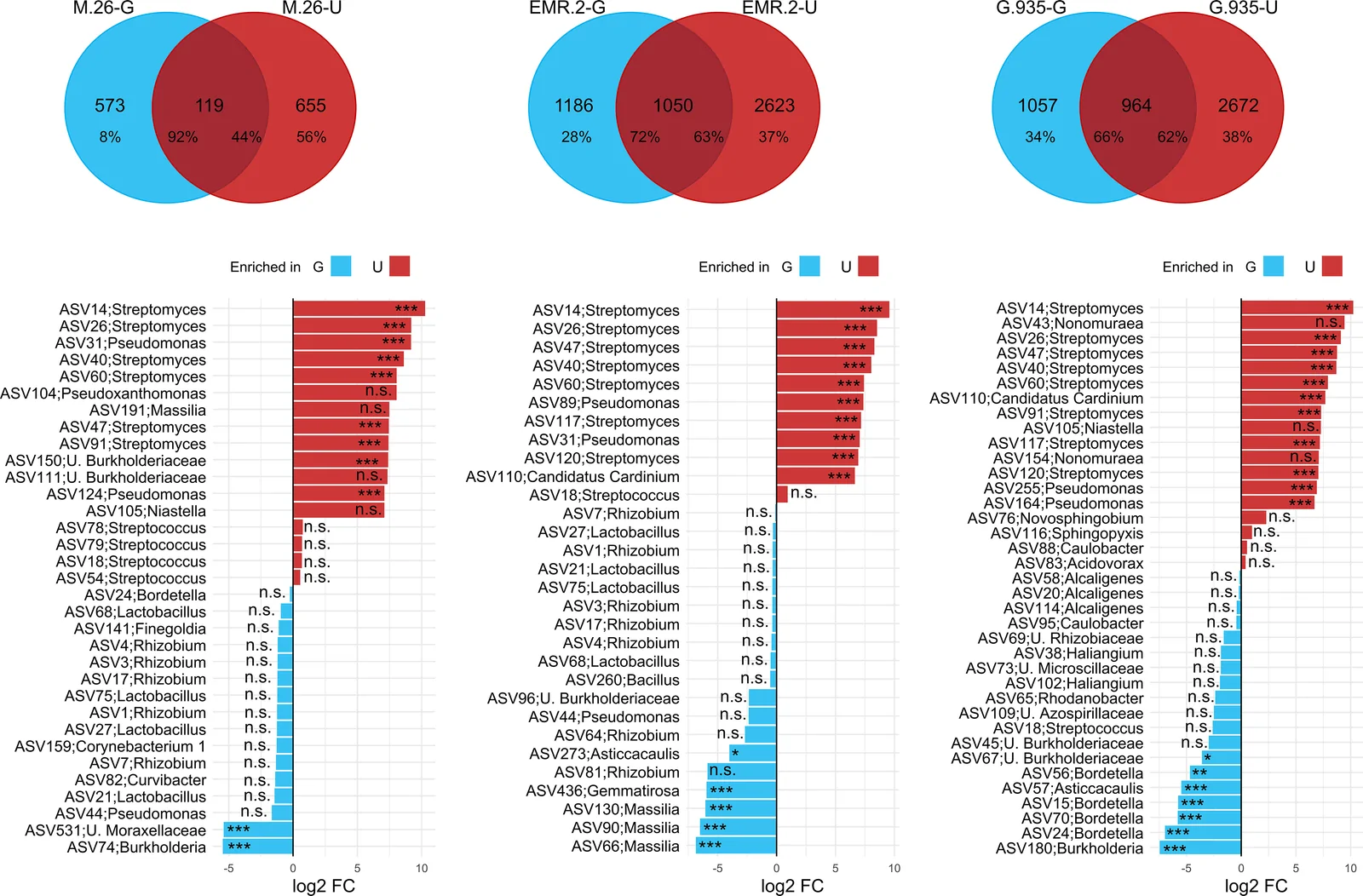
Venn-Diagrams comparing the numbers and relative abundance (RA, %) of endophytic bacteria at ASV level in roots from gamma-irradiated ARD soil (G) and untreated ARD soil (U) of each rootstock genotype (M.26, EMR.2 and G.935) separately. The percentages are aligned towards the treatment they belong to, i.e. G towards left and U towards right, and show the contribution of each compartment to the total RA. Below, log2 fold changes (log2 FC) of the top 20 ASVs of G and U for each of the three rootstock genotypes are displayed (DESeq2; *n* = 5–6; ‘*p ≤ 0.05’, ‘**p ≤ 0.01’, ‘***p ≤ 0.001)

Compared with the more even RA distribution in the rhizosphere, root endophytic communities were dominated by a few highly abundant ASVs (Fig. 6, Suppl. Table 5). Fewer ASVs differed significantly between G and U treatments, while stronger differences were observed among genotypes. In M.26 roots, 573 ASVs were unique to G (8% RA) and 655 were unique to U (56% RA); 119 ASVs were shared between both treatments. Five of the most abundant ASVs belonged to *Rhizobium* (ASV1, ASV3, ASV4, ASV7, ASV17), together representing 71.4% of total RA in M.26-G and 30.2% in M.26-U, although none differed significantly. Among the top 20 ASVs, two (*Burkholderia*, ASV74; unclassified *Moraxellaceae*, ASV531) were unique to G, whereas 12 - including multiple *Streptomyces* and *Pseudomonas* ASVs - were unique to U. All *Streptomyces* and *Pseudomonas* ASVs were significantly more abundant in M.26-U than in M.26-G.

In EMR.2, 1,186 ASVs were unique to G (28% RA), 2,623 to U (37% RA), and 1,050 were shared between both treatments (72% and 63% RA, respectively). Similar to M.26, *Rhizobium* ASVs (ASV1, ASV3, ASV4, ASV7, ASV17, ASV64) dominated the community, comprising 65.5% and 51.0% of total RA in G and U, respectively. Within the top 20 ASVs, *Massilia* (ASV66, ASV90, ASV130), *Rhizobium* (ASV81), *Gemmatirosa* (ASV436), and *Bacillus* (ASV260) were detected exclusively in EMR.2 roots; all ASVs except *Bacillus* were unique to G. As in M.26, several *Streptomyces* (0.7–5.7% RA) and *Pseudomonas* (1.0–1.2% RA) ASVs were unique to U.

In G.935, several ASVs were exclusive to this genotype, including *Haliangium* (ASV38, ASV102), unclassified *Burkholderiaceae* (ASV45), *Bordetella* (ASV70), unclassified *Microspirillaceae* (ASV73), *Novosphingobium* (ASV76), *Caulobacter* (ASV95), unclassified *Azospirillaceae* (ASV109), *Alcaligenes* (ASV114), and *Nonomuraea* (ASV154). Only one ASV among the top 20, *Burkholderia* (ASV180), was unique to G. Notably, *Bordetella* ASVs (ASV15, ASV24, ASV56, ASV70) were significantly more abundant in G.935-G than in G.935-U, while *Streptomyces* (1.0–9.0% RA) and *Pseudomonas* (0.8–0.9% RA) were, again, more abundant in G.935-U. Unlike the East Malling genotypes, *Rhizobium* ASVs were rare in G.935: the combined RA of all *Rhizobium* ASVs reached only 4.2% in G.935-G and 2.7% in G.935-U.

In summary, M.26 and EMR.2 harboured highly similar bacterial communities in the root endosphere, notably dominated by ASVs assigned to *Rhizobium*, while a different, more evenly distributed set of ASVs was characteristic for G.935. Additionally, ARD induced the presence of highly abundant *Streptomyces* and *Pseudomonas* ASVs regardless of rootstock genotype.

### Absolute abundance of endophytic *Streptomyces*

**Fig. 7:**
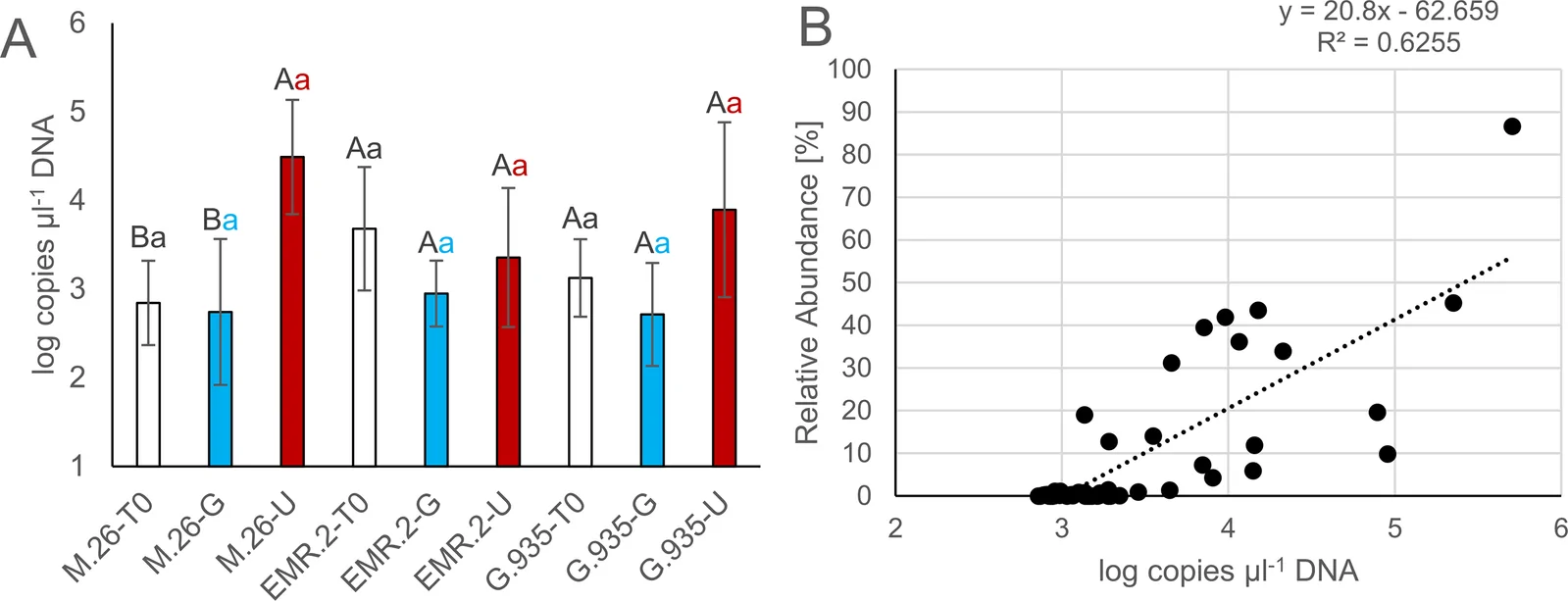
(**A**) Absolute abundance of endophytic *Streptomyces* (log copies µl^− 1^ DNA) for the apple rootstock genotypes M.26, EMR.2, and G.935 before planting (T0) and after 8 weeks of growth in either apple replant disease-affected (U) or gamma-irradiated (G) ARD soil. Differing lower case letters indicate significant differences between genotypes within the same treatment, i.e. T0 (black), G (blue) or U (red). Differing upper case letters indicate significant differences between the treatments within one genotype (lmer, *n* = 6, *p* < 0.05). (**B**) Linear regression of relative abundance (%) and absolute abundance (log copies µl^− 1^ DNA) of endophytic *Streptomyces* (lmtest, *n* = 52, *p* = 3.047e-12)

Since one striking outcome of the amplicon sequencing data was that root endophytic *Streptomyces* were higher in RA by the power of 2–3 in ARD-affected soil in all three rootstock genotypes, we established a qPCR system to quantify *Streptomyces* in apple roots. It revealed a significant 200-fold increase of endophytic *Streptomyces* gene copies in M.26-U compared to M.26-G (Fig. 7a). Increases in EMR.2-U as well as G.935-U were also detected, but not significant. As for the RA data, a high sample variation was observed, which may have hampered the ability to determine statistical differences. A regression between RA via short-read amplicon sequencing and qPCR-based log-transformed absolute quantification data was calculated (Fig. 7b). A significant relationship with an R^2^ of 0.6255 was observed, which was clearly weaker at the lower end of the detected amounts. This coincided with the increased variability that we observed close to the detection limit of the qPCR, i.e. at 35–40 cycles, as 10 copies per µl corresponded to a detection after 34 cycles.

A second qPCR approach targeted plant pathogenic *Streptomyces* based on the *txtAB* gene, which is essential in the biosynthesis of the phytotoxin thaxtomin. While stable results were achieved in the standards, the levels of pathogenic *Streptomyces* remained below the detection limit in all but two samples. Hence, a visualisation of the results was desisted. Overall, the results from the qPCR based analyses confirmed an increase in absolute abundance of endophytic *Streptomyces* in ARD-affected roots but could not deliver evidence supporting plant pathogenicity.

## Discussion

The first question of this study was how ARD and apple rootstock genotypes with differences in ARD susceptibility [34] affect the microbial community composition of the rhizosphere and root endosphere.

For all three analysed genotypes, the α-diversity, which describes the richness in taxa as well as the evenness of their abundance within a sample, in the rhizosphere tended to be higher in ARD soil (U) compared to gamma-irradiated ARD soil (G) (Table 1a). This is in line with previous findings, comparing the bacterial diversity of the apple rhizosphere in ARD soil with that of gamma-irradiated ARD soil [57]. Gamma-irradiation removes almost all organisms from the soil. During eight weeks of cultivation, absent competition likely favoured recolonisation by certain organisms, e.g. those that grow faster or the few that can survive gamma-irradiation, which in turn led to lower diversity in gamma-irradiated soil [58, 59]. Variations in response to the plant genotype existed, but clustering mostly showed differences between G and U (Fig. 2a). Differences in the rhizosphere bacterial community between some apple rootstock genotypes have been observed before [60, 61] and were likely shaped by differences in root exudates.

### Bacterial diversity in the endophytic community might contribute to the reduced ARD sensitivity of the rootstock genotype G.935

In the root endosphere, bacterial α-diversity was lower than in the rhizosphere (Table 1a, b). While no differences between U and G were visible in this microhabitat, the genotype played a more dominant role in shaping the communities with the two East Malling rootstock genotypes, M.26 and EMR.2, having a lower α-diversity (Simpson) than the Geneva rootstock genotype G.935. These observations confirm previous findings [60]. Also, PCoA clustering (Fig. 2a, b) for endophytic bacterial ASVs was both based on treatment (G, U) and rootstock genotype, but the rootstock genotype influence was stronger than in the rhizosphere. Again, M.26 and EMR.2 clustered closely together, separately from G.935.

It is known that plants actively recruit their endophytic microbiome from their environment [62, 63]. While external factors, such as soil type and climate conditions, play an additional role, plants use root exudates to attract and enrich certain microorganisms [62, 63]. Therefore, genotypic differences or similarities, dependent on how closely the genotypes are related, are to be expected. Furthermore, potential tolerance of G.935 against ARD has been postulated by some sources [28, 32, 64], although other studies could not verify clear tolerance towards ARD under their respective testing conditions [34, 61, 65]. Factors, such as the inherent resistance against the putative ARD agent *Pythium ultimum* [66, 67], have previously been discussed to play a role in the postulated tolerance of G.935 against ARD. This can explain the contradicting evaluations of the susceptibility, since *Pythium* sp. might not be involved in the ARD complex in some soils [6]. In our study, oomycetes were not targeted by our analyses. However, other studies that have previously used ARD soil from the same site in Heidgraben could neither find oospores in disease-affected roots nor were able to isolate oomycete strains [2, 68], possibly pointing towards a negligible role of *Pythium ultimum* in ARD soil from this site.

While G.935 showed severe ARD symptoms in the study by Siefen et al. 2024 [34], its ASI was still lower than that of M.26. Aside from specific resistances to pathogens, a higher diversity in the endophytic microbiome could also lead to a more resilient reaction of plants towards detrimental environmental influences. Vandenkoornhuyse et al. (2015) [63] pointed out the possibility of functional redundancy in more diverse microbiomes. This means, specific functionality might not hinge on a single species because it is covered by multiple different taxa. Thus, the higher diversity in G.935 might also contribute to its better performance on ARD-affected soils compared to susceptible rootstock genotypes, such as M.26. For apple, genotypic differences in their root endobiome, especially between East Malling and Geneva rootstock genotypes, were also demonstrated in other recent studies [60, 69, 70]. Moreover, G.935 often stood out having the highest diversity in both endosphere and rhizosphere microbial communities among the tested apple genotypes [61, 71]. Distinct differences in rhizodeposits between M.26 and G.935 were observed and their effect on shaping the microbiome was suggested [72, 73]. It seems likely that the recruitment strategy of G.935 favours a more diverse endobiome, which may have a positive impact on its ability to adapt to external stress, such as ARD. However, this depends on the actual functionality of the endobiome, which remains to be elucidated. In addition, the higher diversity in root endophytic bacteria alone did not explain the susceptibility to ARD, since EMR.2 was also classified as moderately less susceptible than M.26 by Siefen et al. (2024) [34], but its diversity indices were not higher than that of M.26 (Table 1b). This indicates higher diversity alone does not seem to be a sufficient indicator nor cause for reduced ARD susceptibility.

### Possible contribution of *Actinomycetota* to ARD

In both, the rhizosphere and the endosphere of apple roots, *Pseudomonadota* (previously *Proteobacteria*) is usually the dominant phylum [16–18, 22, 74, 75]. We also identified *Pseudomonadota* as the dominant phylum in the endosphere and the rhizosphere. Additionally, a lower RA of *Pseudomonadota* was observed in the rhizosphere of plants from ARD soil (U) compared to plants from gamma-irradiated ARD soil (G). The same trend was observed for the endosphere but the differences were not significant. A reduction of *Pseudomonadota* in ARD-affected soil compared to ARD-unaffected soil was also a common observation in the literature [15, 22, 74, 76].

In both habitats, a significantly higher percentage of *Actinomycetota* was observed in samples from U compared to G regardless of rootstock genotype (Fig. 3a, b; Suppl. Tables 2, 3). Although our observation could partly come from the slower growth style of *Actinomycetota* after gamma-irradiation, the involvement of *Actinomycetota* in ARD has been discussed for decades. Otto et al. (1977) [19] showed a positive correlation between ARD severity and the degree of colonisation of fibrous roots by *Actinomycetes*. Later on, several studies reported increased abundances of *Actinomycetota* in roots from ARD-affected soil compared to healthy soils [2, 20, 22, 74, 77, 78]. Furthermore, Mahnkopp-Dirks et al. (2022) [15] showed that the relative abundance of *Actinomycetota* in roots of apple grown on healthy grassland soil increased over time. However, despite repeated linking of *Actinomycetota* to ARD in the past studies, an active involvement in the disease has neither been discerned nor proven [2].

The phylum *Actinomycetota* harbours many plant beneficial species. Their abilities range from improving nutrient availability and nitrogen fixation over production of plant growth promoting substances to ridding the soil of pollution, e.g. by degrading large hydrocarbons [79]. In the previously published study to our experiment, Siefen et al. (2024) [34] reported strongly increased production of phytoalexins and other phenols in apple roots affected by ARD. Some of these compounds are also known to be exuded into the surrounding soil [80]. Given their ability to tolerate and degrade large hydrocarbons, *Actinomycetota* may be attracted to these compounds and subsequently accumulated in the roots. However, phytoalexin contents in roots and the RA of *Streptomyces* were not correlated, neither total contents nor concentrations of single compounds (results not shown). Inhibition of numerous pathogens due to production of antibiotics by *Actinomycetota*, including the apple fire blight causing bacteria *Erwinia amylovora* [81], have also been shown. However, in past studies on ARD, increased relative abundances of endophytic *Actinomycetota* were usually negatively correlated with growth [19, 20, 22], which seems to contradict an overall positive effect of *Actinomycetota* on apple growth in ARD soil.

*Actinomycetota* also contain a number of plant pathogenic species within the genus of *Streptomyces*. Most well-known is the potato scab causing species *Streptomyces scabies* [82] but other species are also known to cause potato scab [83] or infections of other plants [84, 85]. Due to the wide spectrum of plant beneficial to pathogenic capabilities of *Actinomycetota* species, it is likely that an answer to the question of their involvement in ARD can only be discerned at a lower taxonomic level.

### Rootstock genotype and ARD influenced the rhizosphere and endosphere bacterial communities differently

As described for the diversity data before, when comparing the bacterial community composition in the rhizosphere and root endosphere, the treatment had a stronger effect on the rhizosphere and the influence of the plant host became stronger in the endosphere. While the soil microbiome still significantly affects a plant’s endobiome, plants are able to shape their endophytic microbiome through exudation of a mixture of primary and secondary metabolites, the composition of which depends on a variety of environmental effects [62, 86], but also strongly on the plant genotype, thus driving the recruitment of endophytic microorganisms [62, 87, 88].

As expected, genotypic differences in the rhizosphere barely existed as the bacterial rhizosphere community was mostly shaped by the treatment of the soil the apple plants grew in. The vast majority of ASVs, both in number and RA, was found in all three rootstock genotypes (Fig. 4a). Among the top 20 most abundant ASVs of each rootstock genotype-treatment combination, not a single ASV was unique to one of the three rootstock genotypes (Fig. 5). In G soil, multiple ASVs of the genera *Bordetella* and *Microvirga* appeared in significantly higher relative abundance (RA) compared to U soil for all three rootstock genotypes. Previously proposed ARD-associated taxa such as *Streptomyces*, *Pseudomonas* and *Novosphingobium* [57, 89–91] were not universally higher in the rhizosphere from ARD soil in our case. In fact, among the top 20 ASVs only two *Streptomyces* ASVs were found in higher abundance in EMR.2-U, one in M.26-U and none in G.935-U compared to their respective G variants. No *Pseudomonas* ASVs were found in the top 20 ASVs and a single *Novosphingobium* ASV showed a significantly higher RA in G than U in all three rootstock genotypes. This is not necessarily surprising, as previous reports have not entirely been consistent. Yim et al. (2020) [92] found both *Microvirga* and *Bordetella* to be enriched in gamma-irradiated compared to untreated rose replant disease affected soil but other studies did not find them in significant RA for ARD [57, 89]. Radl et al. (2019) [81] reported an increase in *Novosphingobium* in ARD soil compared to healthy soil, while Hauschild et al. (2024) [90] observed a decrease. *Streptomyces*, while detected in high RA, was not necessarily enriched in ARD-affected rhizosphere [89]. Furthermore, soil origin and a variety of environmental factors before and during the experiment can influence the microbial composition in the rhizosphere. Rumberger et al. (2007) [88] described seasonal shifts of *Pseudomonas* communities so it is possible that the time of year the soil for this experiment was collected influenced the presence of *Pseudomonas* species. Moreover, recolonisation of gamma-irradiated soils will likely depend largely on the prevalent taxa being present in the cultivation surroundings, i.e. irrigation water, greenhouse tables, neighbouring plants etc. Additionally, sequencing techniques can impact the outcome. Lastly, similar functionality might be exercised by different bacteria in different soils.

In the root endosphere, the influence of the soil on the bacterial community became less apparent and genotypic differences became more prevalent. However, a striking enrichment of *Streptomyces* in roots from ARD soil was observed across all rootstock genotypes, with multiple highly abundant ASVs uniquely appearing in roots from U soil (Fig. 6). A similar observation was made for *Pseudomonas* ASVs, although only two ASVs appeared in the top 20 at comparatively lower RA. The observation of highly abundant *Streptomyces* ASVs in ARD-affected apple roots extends similar findings in M.26 [22, 91] to two more apple rootstock genotypes (EMR.2 and G.935) with potentially reduced ARD susceptibility. These results do not support the assumption stated in hypothesis (i) that the accumulation of endophytic *Streptomyces* would be lower in potentially tolerant rootstocks. Mahnkopp-Dirks et al. (2021) [23] observed a negative correlation between growth and several *Streptomyces* ASVs in ARD soil, suggesting a possible involvement of *Streptomyces* in ARD. In this experiment however, no obvious negative correlation between growth and the RA of *Streptomyces* was observed (results not shown). Still, the repeated findings of highly abundant *Streptomyces* in ARD-affected apple roots do suggest an important role, even if not pathogenic.

Genotypic differences were mostly notable between the two East Malling rootstock genotypes M.26 and EMR.2 on the one hand and the Geneva rootstock genotype G.935 on the other hand. ASVs unique to G.935 made up 64% of the total RA in roots from G and 45% in roots from U, while ASVs unique to either M.26 or EMR.2 only added up to a comparatively low RA, respectively. Interestingly, both M.26 and EMR.2 showed very high RA of *Rhizobium* in both U and G (30–71%) but only 3–4% were observed in G.935. Mahnkopp-Dirks et al. (2021) [23] reported an average RA of 4.5% in M.26 roots in field soils pointing to the possibility that *Rhizobium* profited from the conditions in our greenhouse experiment. Other studies, which looked at endophytic bacteria in apple roots from ARD soil mostly did not mention specifically high RA of *Rhizobium* [15, 60]. G.935-G, on the other hand, featured a number of ASVs belonging to the genus *Bordetella* in the top 20 most abundant ASVs, which did not occur in high RA, neither in M.26 nor in EMR.2 roots. Mahnkopp-Dirks et al. (2021) [23] found this genus to be more aligned with healthy roots than ARD-affected roots. However, reports of plant beneficial traits by *Bordetella* are rare [93]. Given the nature of gamma-irradiated soil, we expected the absence of established microbial communities to result in the most competitive organisms to conquer this environment. Our findings of multiple *Bordetella* ASVs among the top 20 most abundant ASVs in the rhizosphere from M.26-G, EMR.2-G and G.935-G support the idea that some *Bordetella* strains are fairly competitive in this environment. The apple plants, however, already carry their established endobiome, which makes the colonisation of the roots more challenging. Additionally, genotypic dependent recruitment strategies significantly influence root colonisation. The differences of highly abundant ASVs in roots of M.26-G and EMR.2-G compared to G.935-G strongly supports the idea, that the genetic origin influences the acquisition process of bacterial endophytes. In ARD-affected (U) roots, the RA of these “genotype-driven” highly abundant ASVs is significantly lower, while the RA of other ASVs, i.e. of the genera *Streptomyces* and *Pseudomonas*, increase regardless of genotype. This suggests that the ability of the plants to steer endophyte acquisition seems to be hampered once roots become damaged.

### Absolute abundance of root endophytic *Streptomyces* suggests no direct detrimental effect

Previous studies have reported a strongly higher RA of endophytic *Streptomyces* in apple roots from ARD soils compared to roots from healthy soils [22, 91]. The results of these studies were solely based on Illumina Miseq sequencing and thus only relied on RA data. While these results likely display an actual increase in the abundance of *Streptomyces*, an overall decrease of the absolute amounts of bacteria could also lead to an observed increased RA of *Streptomyces*. Our qPCR approach aimed to clarify this and showed the absolute abundance of *Streptomyces* to be also elevated (Fig. 7a) in roots from ARD soil compared to roots from gamma-irradiated soil. Due to a high standard deviation, indicating plant-to-plant variation, this difference was only significant for M.26. As also indicated by the positive regression (Fig. 7b), the qPCR reliably mirrored the results of the Illumina Miseq sequencing, especially when the RA of *Streptomyces* was relatively high. However, the relationship was less accurate at low abundance, likely due to two causes: Firstly, the qPCR showed high variability towards the detection limit, i.e. 35–40 cycles, indicating decreased reliability of the results. Secondly, the nature of the rarefaction might disproportionally affect ASVs with close to zero reads.

Due to negative correlations between growth and several *Streptomyces* ASVs, Mahnkopp-Dirks et al. (2021) [23] suggested a possible involvement of *Streptomyces* in the ARD complex. Interestingly among them, one ASV notated as *Streptomyces turgidiscabies*, a well-known causal agent of potato common scab [83]. However, proving factual pathogenicity of *Streptomyces* strains hinges on the ability to retrieve isolates from ARD-affected apple roots. Under lab conditions, *Streptomyces* suffer from a severe competitive disadvantage due to their slow division rate and radial colony growth [94]. Past attempts to isolate *Streptomyces* strains from ARD-affected roots have thus been mostly unsuccessful [15, 21], including numerous attempts at our own lab (unpublished). Tewoldemedhin et al. (2011) [23] managed to isolate a few *Streptomyces* from ARD-affected roots. However, in subsequent pathogenicity tests, none of them turned out to be pathogenic.

In this study, to circumvent the issues surrounding the isolation of strains, we adapted a qPCR method to detect pathogenic *Streptomyces* developed for potato [52]. The qPCR is targeting the *txtAB* gene that codes for the phytotoxin thaxtomin, which is produced by scab-causing *Streptomyces* strains, including *Streptomyces turgidiscabies* [83]. Phytotoxicity of thaxtomin towards other plants has also been demonstrated [95, 96] and *txtAB* was reported to be the most reliable indicator for pathogenicity of a *Streptomyces* strain [52, 97, 98]. Wanner et al. 2009 [99] presented an extensive list of over 100 *Streptomyces* strains, which featured a 100% correlation between the presence of *txtAB* and plant pathogenicity. Therefore, an increase of thaxtomin-producing *Streptomyces* in ARD-affected apple roots would have strongly hinted towards their involvement in ARD as pathogens. However, our results showed no amounts of *txtAB*-carrying *Streptomyces* in ARD-affected apple roots above detection limit. Considering the low detection limit of 1 copy per µL DNA, our results do not support our original assumption stated in hypothesis (ii). This does not completely rule out the possible pathogenicity of the present *Streptomyces* entirely as other genetic targets, which can be used to determine pathogenicity in *Streptomyces*, such as the *Nec1* gene, exist. Furthermore, some studies have reported on plant pathogenic *Streptomyces* strains that do not produce thaxtomins [100, 101]. Still, considering the rarity of such reports and the fact that plant pathogenicity is closely tied to the presence of *txtAB* in the vast majority of cases, our results clearly support the argument against direct pathogenicity. At this point, it needs to be considered that the *Streptomyces* present in ARD-affected apple roots are of saprophytic nature and opportunistically colonise damaged root tissue. However, the possibility of indirect damage, e.g. by suppressing the plant’s immune system to promote mycorrhizal colonisation [102], cannot be ruled out as this could also lead to ease the entry for pathogens as discussed by Mahnkopp-Dirks et al. (2021) [22]. Thus, based on our results, we were only able to partially answer our second research question concerning the role of root endophytic *Streptomyces* in ARD.

## Conclusions

In the current study, the composition of bacterial communities in the rhizosphere was mostly shaped by the soil, rather than rootstock genotype. In the root endosphere, the influence of the rootstock genotype became more apparent. Here, the Geneva rootstock G.935 showed a more diverse and distinct bacterial composition than the two East Malling rootstocks M.26 and EMR.2, which were more similar to each other. The bacterial community of G.935 might help this rootstock genotype to cope with ARD. However, EMR.2 was previously shown to be moderately less susceptible to ARD than M.26, suggesting that other factors could be at play and/or contribute. The results from this study do not suffice for a final conclusion in this regard. For a better understanding of the reasons of reduced ARD susceptibility, more divergent rootstock genotypes and more soils are needed in future studies. ARD had an influence on both rhizosphere and endosphere communities. A general shift away from *Pseudomonadota* as the sole dominant bacterial phylum towards *Actinomycetota* was observed for all three rootstock genotypes. They may be attracted by the high production and partly exudation of aromatic compounds from apple roots. Especially notable was the increased relative abundance of *Streptomyces* found in the root endosphere of samples from ARD soil compared to gamma-irradiated soil, regardless of rootstock genotype. This increase was confirmed by a qPCR approach established in this study for apple roots. However, an additional qPCR approach to quantify pathogenic *Streptomyces* did not detect them in the root samples, pointing away from pathogenic involvement in ARD and potentially towards opportunistic reasons for their increased abundance or their involvement in modifying the plant immune response.

## Supplementary Information

Below is the link to the electronic supplementary material.

Supplementary Material 1

## Data Availability

ASV tables with taxonomic assignments and rarefied reads as well as qPCR data has been uploaded to the BonaRes Repository: https://doi.org/10.20387/bonares-r5cg-rx85. Raw fastq-files from amplicon sequencing have been made available via SRA (NCBI) with the following accession numbers: PRJNA1428594 (rhizosphere bacteria), PRJNA1427620 (endophytic bacteria) and PRJNA1428565 (endophytic fungi).
