# Supplementary material for: Linking microbial community composition of three rootstock genotypes to apple replant disease susceptibility
Source: Environ Microbiome. 2026 Sep 8;21:107. doi: 10.1186/s40793-026-00957-w (PMC13563949; doi:10.1186/s40793-026-00957-w)
Supplement: Supplementary file 1 [file 40793_2026_957_MOESM1_ESM.docx]

Supplementary Material

Supplementary Figure 1. Rarefaction curves of rhizosphere bacterial amplicon sequencing data, displaying species saturation against sequencing depth of A) all samples at a final sequencing depth of 5,321 reads and B) with the lowest read sample (EMR.2-U#2) removed at a final sequencing depth of 14,401 reads. The removal of sample EMR.2-U#2 and subsequently higher sequencing depth led to an increased number of observed species in all samples, leading significant difference between observed species of EMR.2-U compared to EMR.2-G. Statistical significant differences between any variants for the Simpson index and β-diversity were not affected.

Supplementary Table 1. Primers and probes used for qPCR.

| Name | Function | Sequence (5'-3') | Target | Efficiency (%) | R^2^ | Efficiency Inhib. (%) | R^2^ (Inhib.) | Reference |
| --- | --- | --- | --- | --- | --- | --- | --- | --- |
| SMfw8 | Forward-Primer | GCC GAT TGT GGT GAA GTG GA | 23S | 109.7 | 0.995 | 108.4 | 0.975 | Rintala & Nevalinen (2006) [51] |
| SMrev9 | Reverse-Primer | GTA CGG GCC GCC ATG AAA | 23S | 109.7 | 0.995 | 108.4 | 0.975 | Rintala & Nevalinen (2006) [51] |
| SMp6 | Probe | FAM-ATC CTA TGC TGT CGA GAA AAG CCT CTA GCG-TAMRA | 23S |  |  |  |  | Rintala & Nevalinen (2006) [51] |
| StrepF | Forward-Primer | GCA GGA CGC TCA CCA GGT AGT | *txtAB* | 96.4 | 0.997 | 104.3 | 0.988 | Qu et al. (2008) [52] |
| StrepR | Reverse-Primer | ACT TCG ACA CCG TTG TCC TCAA | *txtAB* | 96.4 | 0.997 | 104.3 | 0.988 | Qu et al. (2008) [52] |

Supplementary Table 2. Mean relative abundance (RA, %) and standard deviation of rhizosphere bacteria at phylum level (RA >1%, 100% frequency) for the apple rootstock genotypes M.26, EMR.2, and G.935 before planting (T0) and after 8 weeks of growth in either apple replant disease-affected (U) or gamma-irradiated (G) ARD soil. Differing lower case letters indicate significant differences between genotypes within the same treatment, i.e. G (blue) or U (red). Differing upper case letters indicate significant differences between the treatments within one genotype, i.e. M.26, EMR.2 or G.935 (DESeq2, n=5-6, p<0.05).

| Phylum | M.26-G | M.26-U | EMR.2-G | EMR.2-U | G.935-G | G.935-U |
| --- | --- | --- | --- | --- | --- | --- |
| Pseudomonadota | 48.6±1.1^Aa^ | 25.1±1.1^Ba^ | 39.1±0.5^Aa^ | 33.9±2.0^Ba^ | 34.7±0.8^Aa^ | 26.2±0.8^Ba^ |
| Actinomycetota | 8.0±1.1^Ba^ | 27.5±6.4^Aa^ | 10.7±1.9^Ba^ | 22.6±5.4^Aa^ | 8.8±1.7^Ba^ | 25.1±1.8^Aa^ |
| Chloroflexota | 13.6±0.1^Aa^ | 11.8±0.1^Aab^ | 12.9±0.3^Aa^ | 8.3±0.1^Bb^ | 19.0±0.4^Aa^ | 12.1±0.1^Ba^ |
| Bacillota | 6.5±0.8^Aa^ | 6.9±0.7^Aa^ | 7.1±0.4^Aa^ | 6.3±1.1^Aa^ | 6.5±0.4^Aa^ | 6.9±0.2^Aa^ |
| Acidobacteriota | 2.6±6.0^Ba^ | 9.4±1.5^Aab^ | 2.7±5.3^Ba^ | 8.9±2.3^Ab^ | 2.9±3.4^Ba^ | 11.6±1.1^Aa^ |
| Gemmatimonadota | 4.5±0.3^Ab^ | 3.0±0.0^Ba^ | 5.9±2.2^Aab^ | 2.5±0.0^Ba^ | 7.3±1.0^Aa^ | 2.8±0.0^Ba^ |
| Deinococcota | 0.6±2.0^Aa^ | 0.0±1.8^Ba^ | 7.7±1.8^Aa^ | 0.0±1.6^Aa^ | 4.4±2.1^Aa^ | 0.0±0.9^Aa^ |
| Planctomycetota | 2.8±1.9^Aa^ | 5.3±0.7^Aa^ | 2.4±2.2^Aa^ | 4.4±0.3^Aa^ | 3.6±1.2^Aa^ | 3.9±0.3^Aa^ |
| Verrucomicrobiota | 3.1±1.5^Ba^ | 3.4±0.5^Aa^ | 2.1±1.1^Aa^ | 3.5±0.6^Aa^ | 3.5±1.3^Aa^ | 3.1±0.3^Aa^ |
| Bacteroidota | 2.7±1.1^Ab^ | 1.6±1.2^Ba^ | 2.4±0.4^Ba^ | 2.8±0.5^Aa^ | 2.8±0.9^Aa^ | 2.0±0.4^Ba^ |
| Patescibacteria | 3.0±10.4^Aa^ | 1.1±6.0^Ba^ | 3.4±7.4^Aa^ | 1.7±7.8^Ba^ | 1.8±1.5^Aa^ | 1.0±3.5^Aa^ |
| Armatimonadota | 1.5±0.8^Ab^ | 0.6±0.5^Bab^ | 1.7±0.4^Aab^ | 0.4±0.7^Bb^ | 2.4±1.2^Aa^ | 0.6±0.3^Ba^ |

Supplementary Table 3. Mean relative abundance (RA, %) and standard deviation of endophytic bacteria at phylum level (RA >1%, 100% frequency) for the apple rootstock genotypes M.26, EMR.2, and G.935 before planting (T0) and after 8 weeks of growth in either apple replant disease-affected (U) or gamma-irradiated (G) ARD soil. Differing lower case letters indicate significant differences between genotypes within the same treatment, i.e. T0 (black), G (blue) or U (red). Differing upper case letters indicate significant differences between the treatments within one genotype, i.e. M.26, EMR.2 or G.935 (DESeq2, n=5-6, p<0.05).

| Mean | M.26 -T0 | M.26-G | M.26-U | EMR.2 -T0 | EMR.2-G | EMR.2-U | G.935 -T0 | G.935-G | G.935-U |
| --- | --- | --- | --- | --- | --- | --- | --- | --- | --- |
| Pseudomonadota | 85.4±9.3^a^ | 81.2±21.7^Aa^ | 62±17.3^Aa^ | 84.0±18.4^a^ | 81.0±23.6^Aa^ | 63.8±30.7^Aab^ | 88.5±13.2^a^ | 78.3±10.2^Ab^ | 55.4±26.1^Ab^ |
| Actinomycetota | 6.0±3.4^a^ | 3.1±3.1^Aa^ | 23.8±16.8^Aa^ | 9.7±16.3^a^ | 2.7±3.4^Aa^ | 19.8±17.0^Aa^ | 7.0±12.7^a^ | 4.4±1.8^Ba^ | 33.2±29.7^Aa^ |
| Bacillota | 4.3±6.1^a^ | 14.5±17.7^Aa^ | 9.8±12.0^Aab^ | 2.9±4.2^a^ | 13.9±20.0^Aa^ | 13.5±27.2^Aa^ | 1.3±1.3^a^ | 7.8±6.7^Aa^ | 3.4±2.3^Ab^ |
| Bacteroidota | 3.4±2.3^a^ | 0.6±0.6^Bb^ | 4.1±4.5^Aa^ | 2.2±2.5^a^ | 1.0±0.6^Ab^ | 1.5±0.6^Aa^ | 2.7±1.3^a^ | 6.9±4.5^Aa^ | 6.8±2.4^Aa^ |

Supplementary Table 4. Relative abundance (RA) in G and U as well as significant differences (Sig.; DESeq2; n=5-6; ‘*p≤0.05’, ‘**p≤0.01’, ‘***p≤0.001) of the top 20 rhizosphere bacteria ASVs of G and U for each of the three rootstock genotypes. ASVs unique to G are marked as light blue, unique to U are marked as dark red and unique to the respective rootstock genotype are marked as bold.

Supplementary Table 5. Relative abundance (RA) in G and U as well as significant differences (Sig.; DESeq2; n=5-6; ‘*p≤0.05’, ‘**p≤0.01’, ‘***p≤0.001) of the top 20 bacterial endophyte ASVs of G and U for each of the three rootstock genotypes. ASVs unique to G are marked as light blue, unique to U are marked as dark red and unique to the respective rootstock genotype are marked as bold.

**Supplementary Material: Comprehensive qPCR Material & Methods**

For the quantification of root endophytic *Streptomyces*, each PCR reaction contained 1.25 µL Standard Taq Rection Buffer (B9014, New England Biolabs, Ipswich, USA), 1.25 µL dNTPs (2 mM), 1.25 µL MgCl_2_ (25 mM), 0.5 µL DMSO (Carl Roth, Karlsruhe, Germany), 1.25 µL BSA (2µg/µL, Carl Roth, Karlsruhe, Germany), 1 µL SFfw8 (5 pmol, Suppl. Tab. 1), 1 µL SMrev9 (5 pmol, Suppl. Tab. 1), 0.5 µL SMp6 (5 pmol, Suppl. Tab. 1), 0.0675 µL Hot Start Taq Polymerase (5 U/µL, M0495, New England Biolabs, Ipswich, USA) and 1 µL DNA (1:5 dilution of cleaned-up DNA) in a total volume of 10µL. For the quantification of *Streptomyces*-associated *23S rRNA* gene copies, a positive standard containing the 71 bp target fragment amplified from genomic DNA of *Streptomyces pulveraceus* ES16 was created by cloning. For this, the abovementioned protocol was performed as standard PCR, the PCR product was loaded on a 2 % agarose gel and one band of the correct size (71 bp) was cut from the gel. Subsequently, DNA of the target fragment was extracted using a gel extraction kit (QIAquick Gel Extraction Kit, Qiagen, Hilden, Germany) and the obtained DNA extract was cloned into vector pGEM-T Easy (Promega, Madison, WI, USA). The size of the vector including the target fragment (3071 bp) was checked on a 1 % agarose gel and the plasmid DNA was transformed into competent cells of strain *E. coli* JM109 (Promega, Madison, WI, USA). Positive transformants were determined by Lac-operon-based blue/white selection. Plasmid DNA was extracted from positive transformants and DNA concentration was determined spectrophotometrically (Biophotometer Plus UV/VIS, Eppendorf SE, Hamburg, Germany). The 3071 bp plasmid was isolated from a liquid overnight culture of the transformed *E. coli* in LB-Medium with 100 mg/L ampicillin, using the Plasmid Purification Kit (ThermoScientific, Waltham, USA) according to the manufacturer’s instructions. A dilution series of the plasmid DNA was made (10^0^ to 10^-7^ in 1:10 steps). An endophytic *Novosphingobium sp.*, previously isolated from apple roots, was cultivated in 523 medium [104] for 2 d (24 °C, 150 rpm) and its DNA was extracted for use as a negative control with the following protocol: A cell pellet from 1 mL suspension was mixed with 186.6 µL buffer 1 (50 mM Tris-Cl pH 8.0, 50 mM EDTA pH 8.0, 0.5% Tween 20, 0.5% Triton X-100), 4 µL lysozym (2 mg/L, Carl Roth, Karlsruhe, Germany), 9 µL protease (0.9 AU, Carl Roth, Karlsruhe, Germany) and 0.4 µl RNAse A (200 µg/mL, Carl Roth, Karlsruhe, Germany)) and incubated for 30 min at 37 °C and 900 rpm. Then, 70 µL buffer 2 (3M guanidinhydrochlorid / 20% Tween 20) was added, the suspension vortexed and placed back onto the incubator (30 min, 50 °C, 900 rpm). The cell lysate was gently mixed with 500 µL Binding Solution and 5 µL Silica Powder Suspension (Silica Bead DNA Gel Extraction Kit, Thermo Fisher Scientific, Waltham, USA), incubated for 5 min at 55 °C and inverted manually every 2 min. After centrifugation (5 s, >14,000 *g*), the supernatant was discarded, resuspended in 500 µL Washing Buffer (Silica Bead DNA Gel Extraction Kit) and centrifuged again. This washing step was repeated thrice. After removing the last supernatant, the pellet was dried for 10 min. The pellet was resuspended in 100 µL Tris-HCl (10 mM pH 8.5), incubated (5 min, 55 °C, 900 rpm) and centrifuged (5 s, >14,000 *g*). 50 µL of the supernatant was transferred into a new tube and DNA clean-up was performed with the Monarch PCR & DNA Cleanup Kit (New England Biolabs, Ipswich, USA) according to the manufacturer’s instructions. The potential inhibition of the PCR by residual secondary metabolites in the gDNA from apple roots was tested as follows: A DNA-pool was created by mixing 2 µL of diluted (1:5) DNA of each sample from our experiment. Aliquots of the plasmid dilution series were mixed 1:1 with DNA from the DNA-pool. A qPCR with the following settings was performed with the mixed dilution series as well as the plain dilution series. Primer efficiency was calculated by the CFX Maestro software version 2.3 (Bio-Rad, Hercules, USA) and remained relatively even for both dilution series and remained within the accepted frame of 90-110% (Suppl. Tab. 1).

For the quantification of pathogenic *Streptomyces*, each PCR reaction contained 5 µL SYBR Green iTaq qPCR Mix (Bio-Rad, Hercules, USA), 0.15 µl StrepF (5 pmol, Suppl. Tab. 1), 0.15 µl StrepR (5 pmol, Suppl. Tab. 1), 3.7 µl H2O and 1 µl DNA (1:5 dilution of cleaned-up DNA). A dilution series of *S. scabies* 87.22 (genome size = 10148695 bp, 10^0^ to 10^-7^ in 1:10 steps, 10^0^ ≙ 13.8 ng/µL) was made and the potential inhibition of the PCR by residual secondary metabolites in the gDNA from apple roots was disconfirmed in the same manner as previously described. The standard curve of pure *S. scabies* 87.22 reached detection after 36 PCR cycles at 10^-6^ (~1 copy per µL). Primer efficiency was calculated by the CFX Maestro software version 2.3 (Bio-Rad, Hercules, USA) and ranged within the acceptable frame of 90%-110% (Suppl. Tab. 1).

**Supplementary Material: Analyses of Fungal Endophytes**

**Material and Methods**

DNA used for the analysis of bacterial endophytes was also used for the fungal endophytes. Illumina sequencing was performed by Novogene (Cambridge, UK), using the primer pair ITS-1F-F (CTTGGTCATTTAGAGGAAGTAA) and ITS-1F-R (GCTGCGTTCTTCATCGATGC). Integrity and successful amplification was tested prior to sequencing via gel electrophoresis using company standard procedures. PCR, including a company standard blank, was performed in a total volume of 25 µL (15 ng DNA template, 12.5 µL polymerase, 5 pmol of each primer mix and the following conditions: 15 min at 95 ˚C; 30 cycles of 30 s at 95 ˚C, 30 s at 55 ˚C, 60 s at 72 ˚C; 10 min 72 ˚C. The sequence data was processed in the same manner as the bacterial endophyte data with the following exceptions: 1. The PCR blank did not produce any reads and was not used to prefilter sample reads. 2. Fungal reads were truncated at position 220 (forward) and 220 (reverse), respectively, with an expected error rate of 8. 3. Taxonomic assigned was done using the UNITE 2020-02-20 QIIME release 99%. 4. In the fungal data set, a large number of ASVs was falsely annotated as “unassigned Fungi”. To correct this, the entire dataset was blasted against the nt_core database (NCBI) and 1,763 ASVs not assigned as fungal sequences were removed, leaving 1,386 ASVs assigned as fungi. The core microbiome was only calculated at genus level, with a threshold of 0.5% RA in all samples of one genotype-treatment combination.

**Results**

The richness of endophytic fungi on genus level was relatively low and no significant differences for any of the diversity indices were observed (Suppl. Tab. 6). PERMANOVA (Suppl. Fig. 2) revealed significant effects of treatment, genotype, and their interaction. Three distinct clusters for T0, G, and U were visible in the PCoA (Fig. 2c). Within T0, M.26 clustered slightly separately from EMR.2 and G.935, while within G, EMR.2 clustered slightly separated from M.26 and G.935. Lastly, within U, G.935 clustered slightly separated from M.26 and EMR.2.

Due to a large amount of endophytic fungi without assigned taxa beyond kingdom level, barely any significant differences were found (Suppl. Fig. 3, Suppl. Tab. 7). The RA of *Basidiomycota* was higher in roots of EMR.2-G (12.9±11.5%) compared to roots of EMR.2-U (2.7±5.7%). Roots of G.935 had a higher RA of *Ascomycota* than those of EMR.2 in gamma-irradiated soil (G), 83.7±15.8% compared to 83.2±13.9%, but a significantly lower RA in ARD soil (U), 60.2±13.4% compared to 93.6±8.5%. *Glomeromycota* were significantly higher in abundance in G.935-U (3.9±6.1%) compared to M.26-U (0±0%) roots. Notably, a high proportion of unclassified fungi was found in G.935-U (26.1±19.9%), whereas their abundance remained low in M.26-U (2.3±2.5%) and EMR.2-U (1.1±1.4%).

No significant differences between core root endophytic fungal genera were found between G and U across all genotypes (Suppl. Tab. 8). However, the RA of *Gibberella* was clearly lower in EMR.2-U (0.42±28.09%) compared to EMR.2-G (29.86±35.82%) and in G.935-U (0.33±0.15%) compared to G.935-G (19.06±0.54). Although statistically insignificant, this decrease was also visible in M.26-U (0.26±9.09%) compared to M.26-G (14.94±0.05%). No clear genotypic differences were noticed.

Supplementary Figure 2. β-diversity displayed by Principal Coordinate Analysis (PCoA) of root endophytic fungi for apple rootstock genotypes M.26, EMR.2, and G.935 before planting (T0) and after 8 weeks of growth in either apple replant disease-affected (U) or gamma-irradiated (G) soil. Significant effects of treatment, genotype and their interaction were determined by a PERMANOVA (n=5-6, p<0.05).

Supplementary Figure 3. Relative abundance (RA, %) at phylum level of root endophytic fungi for the apple rootstock genotypes M.26, EMR.2, and G.935 before planting (T0) and after 8 weeks of growth in either apple replant disease-affected (U) or gamma-irradiated (G) soil.

**Discussion**

The α-diversity of endophytic fungi was lower than that of endophytic bacteria. Similar results have been published in other studies [17,60,75]. However, our overall number of observed ASVs was fairly low compared to these sources. We attribute this to the fact, that a high number of reads had to be discarded due amplification of apple sequences. This, in turn, led to lower quality of the whole data set and ultimately resulted in a suboptimal saturation of the rarefaction curve. Due to this, the conclusions on the role of endophytic fungal communities in ARD that can be derived from our data set are compromised and it should be interpreted with care.

For endophytic fungi, *Ascomycota* was the dominant phylum across all genotype-treatment combinations. Its RA ranged from 51.8±18.2% (M.26-T0) to 94.9±3% (M.26-U). Other studies have also reported *Ascomycota* as the dominant fungal phylum in the endosphere with mean relative abundances between 50-60% [10,75]. *Glomeromycota* include many plant beneficial fungi, including arbuscular mycorrhizal fungi, and are often reported to occur in lower abundance in ARD soil compared to healthy grassland soil [10,17,76]. In our study the RA in roots from ARD soil ranged from 0-4% (Suppl. Tab. 6). It needs to be noted, that the used ITS primers do not necessarily cover *Glomeromycota*, especially mycorrhiza, very well. Still, a complete absence of *Glomeromycota* in the gamma-irradiated soil was observed, indicating that gamma-irradiation effectively removed those *Glomeramycota* detected in ARD soil from the soil and subsequent colonisation of the roots did not take place during the duration of our experiment. However, plant growth was significantly better in gamma-irradiated ARD soil [34], so a reduced abundance of *Glomeromycota* might not necessarily hamper plant growth under the supportive growth conditions given.

A number of plant pathogenic fungi have been proposed as putative causal agents of ARD, including *Ilyonectria*, *Dactylonectria*, *Cylindrocarpon*, *Fusarium*, *Rhizoctonia* and *Nectria* [4–10]. Most notably, we did not consistently find any of the proposed fungal pathogens at >0.5% RA in all root samples from ARD soil, regardless of rootstock genotype. Our sample set featured high variability. Additionally, the quality of the data was compromised due to a high number of unspecific apple sequences, which needed to be excluded and subsequently resulted in a mediocre saturation of the rarefaction curve. Hence, we think that no reliable conclusions can be drawn regarding the fungal endophytes based on our results.

Supplementary Table 6. Mean α-diversity indices “Observed” and “Simpson” with standard deviation of endophytic fungi of apple rootstock genotypes M.26, EMR.2, and G.935 before planting (T0) and after 8 weeks of growth in either apple replant disease-affected (U) or gamma-irradiated (G) ARD-affected soil. Differing lower case letters indicate significant differences between genotypes within the same treatment, i.e. T0 (black), G (blue) or U (red). Statistical differences were determined with either Tukey’s test (Observed) or Wilcox’ test (Simpson). Differing upper case letters indicate significant differences between the treatments within one genotype, i.e. M.26, EMR.2 or G.935 (n=5-6, p<0.05).

|  | endophytic fungi | | | | | | | | |
| --- | --- | --- | --- | --- | --- | --- | --- | --- | --- |
|  | M.26-T0 | M.26-G | M.26-U | EMR.2-T0 | EMR.2-G | EMR.2-U | G.935-T0 | G.935-G | G.935-U |
| Observed | 54±9^Aa^ | 58±18^Aa^ | 60±22^Aa^ | 42±11^Aa^ | 41±11^Aa^ | 40±13^Aa^ | 62±17^Aa^ | 65±16^Aa^ | 60±9^Aa^ |
| Simpson | 0.78±0.09^Aa^ | 0.72±0.30^Aa^ | 0.73±0.10^Aa^ | 0.73±0.22^Aa^ | 0.67±0.32^Aa^ | 0.58±0.36^Aa^ | 0.65±0.20^Aa^ | 0.68±0.28^Aa^ | 0.86±0.05^Aa^ |

Supplementary Table 7. Mean relative abundance (RA, %) and standard deviation of endophytic fungi at phylum level (RA >0.5%, 100% frequency) for the apple rootstock genotypes M.26, EMR.2, and G.935 before planting (T0) and after 8 weeks of growth in either apple replant disease-affected (U) or gamma-irradiated (G) ARD soil. Differing lower case letters indicate significant differences between genotypes within the same treatment, i.e. T0 (black), G (blue) or U (red). Differing upper case letters indicate significant differences between the treatments within one genotype, i.e. M.26, EMR.2 or G.935 (DESeq2, n=5-6, p<0.05).

| Mean | M.26-T0 | M.26-G | M.26-U | EMR.2-T0 | EMR.2-G | EMR.2-U | G.935-T0 | G.935-G | G.935-U |
| --- | --- | --- | --- | --- | --- | --- | --- | --- | --- |
| Ascomycota | 51.8±18.2^b^ | 84.3±17.3^Aab^ | 94.9±3.0^Aa^ | 72.4±25.3^a^ | 83.3±13.9^Ab^ | 93.6±8.5^Aa^ | 68.1±27.6^ab^ | 87.3±15.8^Aa^ | 60.2±13.4^Ab^ |
| Basidiomycota | 16.7±7.8^b^ | 13.2±14.1^Aa^ | 2.7±2.5^Aa^ | 15±14.7^b^ | 12.9±11.5^Aa^ | 2.7±5.7^Ba^ | 23.8±30.9^a^ | 10.3±14.3^Aa^ | 9.4±5.9^Aa^ |
| Unclassified_Fungi | 30.8±20.4^a^ | 2.1±3.0^Aa^ | 2.3±2.5^Ab^ | 12.5±20.0^a^ | 3.5±5.5^Aa^ | 1.1±1.4^Ab^ | 7.6±5.9^a^ | 1.9±2.0^Ba^ | 26.1±19.9^Aa^ |
| Glomeromycota | 0.0±0.0^a^ | 0.0±0.0^Ab^ | 0.0±0.0^Ab^ | 0.0±0.1^b^ | 0.0±0.0^Bb^ | 2.6±6.3^Aab^ | 0.0±0.0^ab^ | 0.0±0.0^Aa^ | 3.9±6.1^Aa^ |
| Mortierellomycota | 0.4±0.6^a^ | 0.5±0.6^Aa^ | 0.0±0.1^Aa^ | 0.0±0.0^b^ | 0.2±0.3^Aa^ | 0.0±0.0^Aa^ | 0.1±0.1^ab^ | 0.4±0.3^Aa^ | 0.3±0.2^Aa^ |
| Chytridiomycota | 0.0±0.0 | 0.0±0.0 | 0.0±0.0 | 0.1±0.2 | 0.0±0.0 | 0.0±0.0 | 0.4±1.0 | 0.0±0.0 | 0.0±0.0 |
| Rozellomycota | 0.2±0.5 | 0.0±0.0 | 0.0±0.0 | 0.0±0.0 | 0.0±0.0 | 0.0±0.0 | 0.0±0.0 | 0.0±0.0 | 0.0±0.1 |
| Mucoromycota | 0.0±0.0 | 0.0±0.0 | 0.0±0.0 | 0.0±0.0 | 0.0±0.0 | 0.0±0.0 | 0.0±0.0 | 0.0±0.0 | 0.0±0.0 |

Supplementary Table 8. Mean relative abundance (RA, %) and standard deviation of endophytic fungi at genus level (RA >0.5%, 100% frequency) for the apple rootstock genotypes M.26, EMR.2, and G.935 before planting (T0) and after 8 weeks of growth in either apple replant disease-affected (U) or gamma-irradiated (G) ARD soil. Differing lower case letters indicate significant differences between genotypes within the same treatment, i.e. T0 (black), G (blue) or U (red). Differing upper case letters indicate significant differences between the treatments within one genotype, i.e. M.26, EMR.2 or G.935 (DESeq2, n=5-6, p<0.05).

| Genus | M.26-T0 | M.26-G | M.26-U | EMR.2-T0 | EMR.2-G | EMR.2-U | G.935-T0 | G.935-G | G.935-U |
| --- | --- | --- | --- | --- | --- | --- | --- | --- | --- |
| *Plectosphaerella* | 31.34±22.63^Aa^ | 02.57±23.15^Bb^ | 08.15±22.72^ABab^ | 22.57±02.76^Aa^ | 04.07±07.48^Bb^ | 01.02±35.67^Bb^ | 30.6±18.57^Aa^ | 29.50±01.39^Aa^ | 23.49±11.14^Aa^ |
| *Gibberella* | 00.02±00.02^Bb^ | 14.94±00.05^Aa^ | 00.26±09.09^Aa^ | 00.04±20.32^Bb^ | 29.86±35.82^Ab^ | 00.42±28.09^Ba^ | 04.37±00.15^Aa^ | 19.06±00.54^Aab^ | 00.33±00.15^Ba^ |
| *Conlarium* | 00.19±00.18^Ab^ | 01.32±11.66^Aa^ | 00.05±25.44^Aab^ | 05.17±02.23^Aa^ | 00.69±01.08^Aa^ | 00.00±01.43^Bb^ | 13.37±00.12^Aa^ | 00.93±00.01^ABa^ | 00.78±00.33^Ba^ |
| *Ceratobasidium* | 07.48±02.88^Aa^ | 00.22±01.70^ABa^ | 00.11±03.05^Ba^ | 01.39±00.35^Aa^ | 00.05±00.09^ABa^ | 00.23±04.83^Ba^ | 02.10±00.12^Aa^ | 02.32±00.50^Aa^ | 00.40±00.66^Aa^ |
| Others | 60.97±21.21 | 70.83±27.89 | 49.56±25.34 | 80.96±18.85 | 65.33±33.59 | 48.19±33.68 | 91.43±18.57 | 98.32±02.20 | 75.00±11.31 |
